# Targeted delivery of glucocerebrosidase to lysosomes: The LYSOTAC (LYSOsome-TArgeting Chimera) technology

**DOI:** 10.1016/j.ajps.2026.101149

**Published:** 2026-03-16

**Authors:** Hee-Yeon Kim, Eun Nam Choi, Gee Eun Lee, Sanghwa Yoon, Su Ran Mun, Eui Jung Jung, Minji Kim, Hyomin Lim, Yang Jae Kang, Woo-Jae Park, Yong Tae Kwon, Joo-Won Park

**Affiliations:** a Department of Biochemistry, College of Medicine, Ewha Womans University, Seoul 07804, Republic of Korea; b Cellular Degradation Biology Center and Department of Biomedical Sciences, College of Medicine, Seoul National University, Seoul 03080, Republic of Korea; c Research Institute of Molecular Alchemy, Gyeongsang National University, Jinju, 52828, Republic of Korea; d Department of Medicine, Columbia University Irving Medical Center, New York, NY 10032, USA; e Division of Bio & Medical Bigdata Department (Brain Korea 21 Four), Gyeongsang National University, Jinju 52828, Republic of Korea; f Division of Life Science Department, Gyeongsang National University, Jinju 52828, Republic of Korea; g Department of Biochemistry, Chung-Ang University College of Medicine, Seoul 06974, Republic of Korea; h AUTOTAC Bio Inc., 225 Gasan digital 1-ro, Geumcheon-gu, Seoul 08501, Republic of Korea; i Ischemic/Hypoxic Disease Institute, College of Medicine, Seoul National University, Seoul 03080, Republic of Korea; j Convergence Dementia Research Center, Seoul National University Medical Research Center, Seoul 03080, Republic of Korea; k National Research Laboratory for Convergence Degradation Biology, Korea University, Seoul 02841, Republic of Korea

**Keywords:** Lysosomal targeting, N-degron pathway, p62/SQSTM1/Sequestosome-1, Autophagy, Gaucher disease, Lysosomal storage diseases

## Abstract

Lysosomal storage diseases (LSDs) are a group of inherited metabolic disorders caused by misfolding of lysosomal proteins and their degradation via endoplasmic reticulum-associated degradation (ERAD). Deficiency in LSD-associated enzymes leads to the accumulation of toxic materials within the lysosome. In macroautophagy (hereafter autophagy), autophagic receptors as represented by p62/SQSTM1/Sequestosome-1 collect and deliver their cargoes to the lysosome. Here, we developed the LYSOTAC (LYSOsome-TArgeting Chimera) technology, which enables lysosomal targeting of LSD-associated enzymes while preserving their enzymatic activities. LYSOTAC employs a bifunctional chimera that simultaneously binds an LSD-associated enzyme via the enzyme-binding ligand (EBL) and p62 via the autophagy-targeting ligand (ATL). Upon binding, p62 undergoes self-polymerization to form cargo-p62 complexes, which are sequestered into autophagosomes and delivered to lysosomes, where the enzymes exhibit maximal activity. Here, LYSOTAC compounds targeting β-glucocerebrosidase (GCase) were designed to restore GCase activity in lysosomes and promote glucosylceramide degradation in Gaucher disease fibroblasts. We suggest that LYSOTAC provides a potential therapeutic strategy for LSDs.

## Graphical abstract

**Figure:**
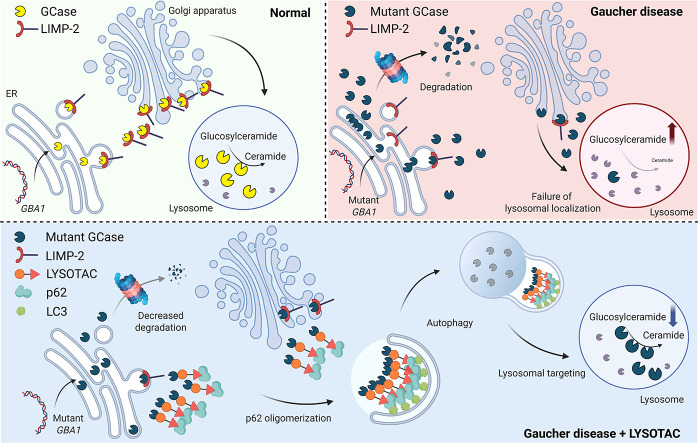

## 1 Introduction

Lysosomal storage diseases (LSDs) refer to a group of over 70 metabolic diseases caused by malfunctioning enzymes whose activity is required to degrade their substrates within the lysosome. The accumulation of undegraded substrates including lipids, glycoproteins, and mucopolysaccharides in the lysosome leads to lysosomal dysfunction, resulting in a variety of clinical manifestations depending on the substrates and damaged organs. LSDs exhibit the prevalence of 1 in 5000 live births [1] and are accompanied by genetic mutations of the proteins involved in lysosomal functions. Mutations in lysosomal enzymes occasionally cause misfolding and impaired trafficking, leading to endoplasmic reticulum-associated degradation (ERAD).

Gaucher disease (GD) is a prominent LSD among Ashkenazi Jews, with a prevalence of 1 per 450 births [2] versus 0.9 per 100,000 in the general population (95% confidence interval 0.7–1.1) [3]. GD is inherited as an autosomal recessive trait with mutations in the *GBA1* gene, which encodes β- glucocerebrosidase (GCase). A defective GCase causes the accumulation of glucosylceramide, also called glucocerebroside, leading to various clinical manifestations including hepatosplenomegaly and bone abnormalities as defined by type 1 GD. Although relatively rare, type 2 and 3 GD manifest neurological symptoms. The most prevalent mutation in GCase is N409S (previously called N370S) associated with type 1 GD [4]. Severe variants (e.g., L483P, previously called L444P) almost completely inactivate lysosomal GCase and are more likely to lead to type 2 and 3 GD [4]. These GCase mutants are prone to misfolding during maturation in the endoplasmic reticulum (ER) and trafficking through the Golgi body to the lysosome. Misfolded GCase is recognized by ER quality control machinery, leading to proteasome degradation via ERAD, resulting in the deficiency of GCase in the lysosome [4]. The hydrolytic activity of GCase is maximally active at pH 5.5 in the lysosomal compartment [5]. Following a folding process, GCase arrives at the lysosome exclusively in complex with its receptor lysosomal integral membrane protein 2 (LIMP-2), that facilitates its transport to the lysosome [5].

Current options for the treatment of GD include enzyme replacement therapy (ERT), wherein a recombinant GCase enzyme, such as imiglucerase, is administered intravenously [4]. ERT is very expensive, with annual costs of up to approximately half a million dollars for a 70 kg adult [6]. Moreover, because recombinant enzymes used for ERT do not cross the blood-brain barrier (BBB), ERT can only be applied to type 1 GD, not to type 2 or 3 GD. As an alternative approach, substrate reduction therapy has been explored to reduce the toxic glucosylceramide species by inhibiting glucosylceramide synthase using chemical drugs such as miglustat and eliglustat [4]. In LSDs, many of the mutant forms of lysosomal enzymes retain catalytic activity or exhibit only modestly compromised function despite mild conformational alterations; however, ER quality control prevents their trafficking to lysosomes, leading to ERAD [7]. To rescue the activities of mutant LSD enzymes, pharmacological chaperone therapy (PCT) has been employed, in which small molecules assist the folding of LSD-associated enzymes [8] and, thus, hijack otherwise unstable enzymes from ERAD [7].

The N-degron pathway regulates the degradation of various biomaterials [9,10]. In this process, a structurally distinct N-terminal (Nt-) moiety, called an N-degron, is bound by its cognate N-recognin that link the targeted substrates to the ubiquitin-proteasome system or autophagy. Known N-degrons include the Nt-arginine (Nt-Arg) residue that can be generated by post-translational conjugation of the amino acid L-Arg by arginyl-tRNA–protein transferase (ATE1) [11,12]. We have previously shown that Arg/N-degrons bind p62 to facilitate the lysosomal degradation of proteins and other materials via p62-dependent autophagy [13]. When cells sense autophagic cargoes, Arg/N-degrons are generated on Nt-residues of intracellular proteins and bind the ZZ (ZZ-type zinc finger) domain of p62 [14,15]. Following an allosteric conformational change, p62 undergoes self-polymerization in complex with its associated cargoes, such as misfolded proteins, leading to high-molecular-weight species via liquid-liquid phase separation [16]. In parallel, the exposed LIR (LC3-interacting region) domain of p62 enables binding to LC3-II on phagophores [17]. This N-degron principle has been shown to mediate the lysosomal degradation of proteins and subcellular organelles, as well as intracellular bacteria [[18], [19], [20], [21], [22], [23]]. Based on this allosteric mode of action, we also developed chemical mimetics of Arg/N-degrons, termed autophagy-targeting ligands (ATLs), that can activate p62 in autophagic targeting of various cargoes [24].

We previously reported the AUTOTAC (AUTOphagy-TArgeting Chimera) technology that employs bifunctional molecules in which target-binding ligands (TBLs) are linked to ATLs [[25], [26], [27], [28]]. This technology leads to lysosomal degradation of targets via ATL-mediated p62 activation. In the current study, we utilized AUTOTAC to target lysosomal enzymes of LSDs to the lysosome, wherein cargoes complexes associated with p62 are dissociated into functional monomeric enzymes. This technology, termed LYSOTAC (LYSOsome-TArgeting Chimera), is similar with AUTOTAC in a bifunctional configuration containing the ATL but different from AUTOTAC in physiological outcomes: loss-of-function versus gain-of-function. As a proof-of-concept, we designed two LYSOTAC compounds (ATB2057 and ATB2058), in which GCase is recognized by an enzyme-binding ligand (EBL) which in turn is linked to an ATL. We show that these LYSOTAC compounds facilitated the lysosomal targeting of GCase in GD fibroblasts. The lysosome-targeted GCase complexes were dissociated into at least partially active enzymes. Our data identify a novel lysosome-targeting strategy based on p62-mediated selective autophagy, which provides therapeutic insights as either a primary or an adjuvant treatment for LSDs.

## 2 Materials and methods

### 2.1 Materials

JZ-4109 was synthesized as reported previously [29]. ATB1021 ((R)-1-(3,4-bis((4-fluorobenzyl)oxy)phenoxy)-3-((2-hydroxyethyl)amino)-propan-2-ol), also called YT-6-2, was synthesized as described [21]. All the chemicals were purchased from Sigma-Aldrich (St. Louis, MO, USA) unless otherwise stated. The antibodies used were as follows: mouse monoclonal anti-p62 (Abcam, Cambridge, UK, ab56416) for immunocytochemistry (ICC) and Western blotting (WB); mouse monoclonal anti-GCase (Sigma-Aldrich, WH0002629M1) for WB; rabbit polyclonal anti-p62 (Cell Signaling Technology, Danvers, MA, USA, 23214) for ICC; rabbit polyclonal anti-LC3 (Sigma-Aldrich, L7543), rabbit monoclonal anti-LAMP1 (Cell Signaling Technology, 9091), rabbit monoclonal anti-LIMP2 (Abcam, ab176317), rabbit polyclonal anti-β-actin (Bioworld Technology, St. Louis Park, MN, USA, AP0060), and goat polyclonal anti-glyceraldehyde 3-phosphate dehydrogenase (GAPDH; GenScript, Piscataway, NJ, USA, A00191). Mouse monoclonal anti-GCase for ICC (clone 1/17) [30] was generously provided by The Michael J. Fox Foundation for Parkinson's Research (New York, NY, USA) and is originally sourced from F. Hoffmann-La Roche Ltd (Basel, Switzerland). The following secondary antibodies were used: Alexa Fluor 488 goat anti-rabbit IgG (Invitrogen, A11034), Alexa Fluor 488 goat anti-mouse IgG (Invitrogen, A11029), Alexa Fluor 555 goat anti-rabbit IgG (Invitrogen, A32732), Alexa Fluor 555 goat anti-mouse IgG (Invitrogen, A32727), Alexa Fluor 647 goat anti-mouse IgG (Invitrogen, A21235), anti-rabbit IgG-HRP (Cell Signaling Technology, 7074), and anti-mouse IgG-HRP (Cell Signaling Technology, 7076). LysoTracker (L7528) was purchased from Thermo Fisher Scientific (Waltham, MA, USA).

### 2.2 Chemical synthesis of LYSOTAC compounds

Detailed information on the synthesis of LYSOTAC compounds is provided in the Supplementary materials.

### 2.3 Human cells

HEK293 and HeLa cells were obtained from the American Type Culture Collection (ATCC, Manassas, VA, USA) and cultured in Dulbecco’s modified Eagle’s medium (DMEM; Gibco) supplemented with 10% fetal bovine serum (FBS; Gibco) and 1% penicillin-streptomycin (10,000 U/ml; Gibco). Human fibroblasts, including GM08680 (wild-type (WT) *GBA1*), GM05381 (WT *GBA1*), GM02627 (*GBA1* G364R/C381G), GM00372 (*GBA1* N409S/L29Afs*18) and GM07968 (*GBA1* L483P/L483P), were acquired from Coriell Biorepositories (Camden, NJ, USA) and cultured in Eagle's minimum essential medium containing Earle's salts (MEM, Gibco), non-essential amino acids (Gibco), and 2 mM L-glutamine supplemented with 15% FBS. The fibroblasts used for the experiments were at approximately passage 11–20. To perform an autophagic flux assay, cells were treated with E64d (10 µg/ml, 24 h) and pepstatin A (PA, 10 µg/ml, 24 h). To evaluate the ability of the compounds to restore GCase protein levels and enzymatic activity, cells were treated for 10 d, following the conditions described in a previous study [31].

### 2.4 Generation of SCARB2^-/-^ HEK293 cell line

LIMP-2-deficient HEK293 cells were produced using a LIMPII (SCARB2) Human Gene Knockout Kit (CRISPR) (#KN402162; Origene, Rockville, MD, USA) according to the manufacturer’s instructions. Briefly, HEK293 cells with 50% confluence were transfected with a guide RNA (gRNA) vector using TurboFectin 8.0. The cells were subcultured for 2 weeks and selected with the medium containing puromycin (1 µg/ml). Puromycin-resistant cells were further processed with limited dilution to isolate individual cell colonies.

### 2.5 Molecular docking simulation

The crystal structure of the ZZ domain of human p62 (PDB ID: 7R1O) was obtained from the RCSB Protein Data Bank [32]. To refine the crystal structure, hydrogen atoms were added and protonation states were assigned at physiological pH (7.0) using the *Clean Protein* tool in Discovery Studio (DS) 2022 (BIOVIA, San Diego, CA, USA). The two-dimensional (2D) structure of ATB1021 was generated using MarvinSketch software and converted into a three-dimensional (3D) conformation. Geometry optimization was conducted through the *Minimize ligands* tool with the CHARMm force field in DS. A total of 2000 steps were run using the smart minimizer algorithm under implicit solvation conditions modeled by the Generalized Born Molecular Volume (GBMV) method. Molecular docking was carried out using the Genetically Optimized Ligand Docking (GOLD) Suite version 5.2.2 (Cambridge Crystallographic Data Centre, Cambridge, UK), which employs a genetic algorithm to explore ligand conformations [33]. The binding site was defined as a spherical region with a 10 Å radius centered on the co-crystallized ligand in the 7R1O structure. Docking was repeated 10 times under default parameters. RMSD-based clustering with a cutoff of 2.5 Å was employed to analyze the docking poses. The representative binding conformation was selected as the pose with the highest GOLD docking score within the most populated cluster.

### 2.6 Construction of 3D model for LYSOTAC ternary complex

The crystal structures of GCase in complex with JZ-4109 (PDB ID: 5LVX) and the ZZ domain of human p62 (PDB ID: 7R1O) were obtained from the RCSB Protein Data Bank [29]. The GCase–JZ-4109 complex was utilized without additional docking, as the ligand was co-crystallized in the binding site. To construct the complete LYSOTAC molecule, JZ-4109 and ATB1021 were manually linked via a polyethylene glycol (PEG)-based linker using MarvinSketch software. The resulting structure was converted into a 3D conformation and subjected to energy minimization using the *Minimize ligands* tool with the same parameters as described in Section 2.5. The optimized LYSOTAC structure was manually positioned within the binding pockets of GCase and the p62 ZZ domain by aligning each ligand moiety with its respective binding site. To resolve steric hindrance and improve the geometric quality of the complex model, structural refinement was performed using the *Minimization* protocol with the CHARMm force field in DS. The energy minimization was carried out for 10,000 steps using the Steepest Descent algorithm under implicit solvation conditions based on the GBMV model.

### 2.7 Cell viability assay

Cell viability was measured with a 3-(4,5-dimethylthiazol-2-yl)-2,5-diphenyltetrazolium bromide (MTT) assay as described [34]. After cells in a 96-well plate were treated with ATB2057 or ATB2058 for 24 h, MTT solution was added to the medium at a final concentration of 0.5 mg/ml, and then cells were incubated for 4 h at 37 °C in a CO_2_ incubator. The medium was replaced with dimethyl sulfoxide (DMSO), and optical density values were measured at 540 nm.

### 2.8 Transfection

Plasmids and small interfering RNAs (siRNAs) were transfected into cells using TurboFectin 8.0 (Origene) and RNAiMAX Transfection Reagent (Invitrogen), respectively, according to the manufacturer’s protocols.

### 2.9 Immunoblot analysis

Cells were lysed using KPi buffer (25 mM KPi, pH 6.5; 0.1% Triton X-100) containing a protease cocktail (Sigma-Aldrich). After the protein concentration of lysates was determined using a Protein Assay Dye Reagent (Bio-Rad, Hercules, CA, USA), 15–20 µg of each protein was treated with endoglycosidase H (Endo-H) according to the manufacturer’s protocol (New England Biolabs, Ipswich, MA, USA). Samples boiled in Laemmli sample buffer (Bio-Rad) were separated in 6%-15% SDS polyacrylamide gels and transferred to nitrocellulose membranes (Bio-Rad), followed by blocking with 5% bovine serum albumin (BSA; Sigma-Aldrich) in TBST (TBS with 0.1% Tween 20) for 1 h. Blotted membranes were subsequently incubated with primary and secondary antibodies, followed by detection using SuperSignal West Pico Chemiluminescent Substrate (Thermo Fisher Scientific) and the ChemiDoc MP Imaging System (Bio-Rad). Protein bands were subjected to densitometry using ImageJ software (National Institutes of Health [NIH], Bethesda, MD, USA).

### 2.10 Immunocytochemistry (ICC)

Cells were cultured on coverslips coated with collagen (Sigma-Aldrich) and fixed with 4% paraformaldehyde in phosphate buffered saline (PBS) for 10 min, followed by permeabilization with 0.1% Triton X-100 in PBS for 10 min. After washing in PBS, the cells were blocked with 2% bovine serum albumin (BSA) in PBS for 1 h and incubated with primary antibodies diluted in 2% BSA/PBS solution overnight at 4 °C, followed by incubation with Alexa Fluor-conjugated secondary antibodies (Thermo Fisher Scientific). After the coverslips were mounted on glass slides using a mounting medium (Vector Laboratories), images were analyzed using a laser scanning confocal microscope (510 Meta; Zeiss, Oberkochen, Germany) and Zeiss LSM Image Browser (ver. 4.2.0.121).

### 2.11 LC-ESI-MS-MS analysis of glucosylceramide

The cellular levels of glucosylceramide were measured using liquid chromatography-electrospray ionization-tandem mass spectrometry (LC-ESI-MS-MS) as described previously [35,36] with some modifications. Briefly, total lipids extracted from cells (10 µl) were injected into a UHPLC (ExionLC™ Series UHPLC, AB SCIEX, Toronto, Canada) and separated on a reversed-phase Kinetex HILIC column (2.1 mm × 100 mm ID, 2.6 µm; Phenomenex, St. Louis, MO, USA). The column effluent was introduced into an API 5500 QTRAP mass spectrometer (AB SCIEX). Analyses were performed using ESI in positive-ion mode with multiple reaction monitoring (MRM) to select both parent and characteristic daughter ions specific to each analyte simultaneously from a single injection. Data were acquired and processed using Analyst 1.6.3 software (Applied Biosystems, Foster City, CA, USA).

### 2.12 GCase activity assay

The enzymatic activity of GCase was measured as described previously [37] with some modifications. Briefly, cells were harvested in the assay buffer (50 mM citric acid, 176 mM K_2_HPO_4_, pH 5.9; 0.01% Tween 20, 10 mM sodium taurocholate) and incubated on ice for 10 min. After centrifugation at 10,000 × g for 30 min at 4 °C, the supernatant was used for further assay. Ten microliters of the supernatant was added to 40 µl of 5 mM 4-methylumbelliferone-β-D-glucopyranoside (Sigma-Aldrich, #M3633) substrate. After incubation for 30 min at 37 °C, the reaction was terminated using 70 µl stop solution (0.4 M glycine, 1 M sodium hydroxide, pH 10.6). Plates were read (Ex 360/Em 460) using a SpectraMax plate reader (Molecular Devices, Sunnyvale, CA, USA). Enzymatic activity was normalized to total protein content in each sample.

### 2.13 Lysosome fractionation

Lysosome isolation was performed using the Lysosome Isolation Kit from Abcam according to the manufacturer’s protocol [38]. Briefly, 2 × 10^7^ cells were homogenized using a glass Dounce homogenizer in Lysosome Isolation Buffer. After adding Lysosome Enrichment Buffer, the supernatant was collected by centrifugation at 500 × g for 10 min at 4 °C. The supernatant was carefully placed on top of the density gradient solutions, followed by ultracentrifugation at 145,000 × g for 2 h at 4 °C. The lysosome fraction band was carefully extracted from the top of the gradient and resuspended in two volumes of PBS. Finally, the sample was centrifuged at 18,000 × g for 30 min at 4 °C, and the pellet was regarded as the purified lysosomes.

### 2.14 Real-time PCR

Total RNA was extracted from cells using Direct-zol™ RNA MiniPrep kit (Zymo research, Irvine, CA, USA). cDNA was synthesized from isolated ReverTra Ace® qPCR RT Master Mix with gDNA Remover (Toyobo, Osaka, Japan) according to the manufacturer's protocol. Real-time PCR was performed using SYBR green (Thermo Fisher Scientific) in a 7500 Fast Real-Time PCR system (Thermo Fisher Scientific). All expression values were normalized with *HPRT* gene. Following primers were used for real-time PCR: *LAMP1* forward: 5′- CGTGTCACGAAGGCGTTTTCAG-3′, reverse: 5′-CTGTTCTCGTCCAGCAGACACT-3′ [39]; *M6PR* forward: 5′-CACCTCAGTGTGGGTTCCATCT-3′, reverse: 5′-ATCCTGCCAGAAGGCTAAGTGG-3′; *MCOLN1* forward: 5′-CGGACTGCTATACCTTCAGCGT-3′, reverse: 5′-GGTGCTTACACTCCTGGATGTG-3′ [40]; *p62* forward: 5′- CCTCTGGGCATTGAAGTTG-3′, reverse: 5′-ATCCGACTCCATCTGTTCCTC-3′; *HPRT* forward: 5′-ACGTCTTGCTCGAGATGTGA-3′, reverse: 5′-AATCCAGCAGGTCAGCAAAG-3′ [41].

### 2.15 In vitro p62 aggregation assay

HEK293T cells were transfected with p62-myc/hi and lysed with buffer [50 mM HEPES (pH 7.4), 0.15 M KCl, 0.1% Nonidet *p* - 40, 10% glycerol, containing a mixture of protease inhibitors and phosphatase inhibitor (Sigma-Aldrich)]. Lysates were centrifuged at 13,000 × g for 20 min at 4 °C after 10 cycles of freezing and thawing. The supernatants were collected, and 1 µg of total protein was incubated with indicated compounds (1 mM) at room temperature for 2 h. Samples were mixed with 4 × LDS sample buffer under non-reducing condition, resolved by SDS-PAGE, and immunoblotted with an anti-myc antibody.

### 2.16 Quantification and statistical analysis

Statistical significance of differences between various groups was determined by unpaired Student’s *t*-test, one-way analysis of variance (ANOVA), and two-way ANOVA (GraphPad Prism ver.10.4.2, San Diego, CA, USA). The results are expressed as the means ± SEM. Differences were considered significant at *P* < 0.05 (^∗∗∗^*P* < 0.001; ^∗∗^*P* < 0.01; ^∗^*P* < 0.05; N.S., not significant).

## 3 Results and discussion

### 3.1 Development of LYSOTAC targeting GCase

**Fig. 1:**
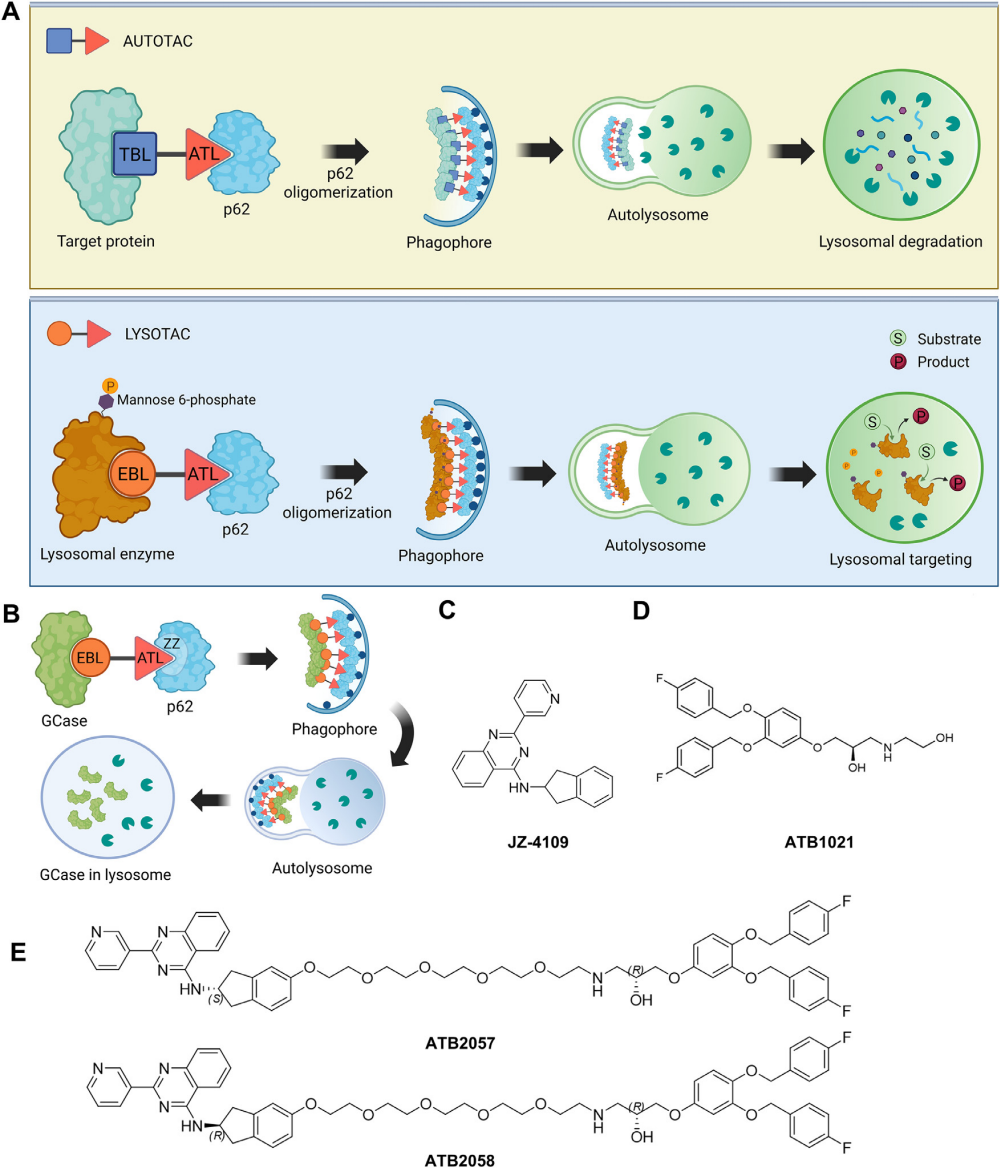
Development of LYSOTAC compounds. **(A)** Schematic diagram of LYSOTAC technology compared to AUTOTAC (Created in BioRender. Park, J. (2026) https://BioRender.com/ijm1ere); **(B)** Schematic diagram of a LYSOTAC compound designed to deliver GCase to lysosomes (Created in BioRender. Park, J. (2026) https://BioRender.com/nxs6jn7); (C-D) Chemical structures of JZ-4109 used as EBL, ATB1021 used as ATL, and ATB2057 and ATB2058 generated as LYSOTAC compounds targeting GCase.

Whereas AUTOTAC induces the degradation of targeted proteins by lysosomal hydrolases, LYSOTAC aims to deliver LSD-associated lysosomal enzymes to the lysosome while maintaining their activity by using p62 as a vehicle (Fig. 1A). A LYSOTAC compound is composed of EBL linked to ATL in a way that does not interfere with the complex formation of p62 associated with its cargo. To select an EBL targeting GCase (Fig. 1B), we evaluated the candidacy of several compounds including isofagomine, *N*‑butyl‑deoxynojirimycin (NN-DNJ), ambroxol and JZ-4109 [42]. Excluded were isofagomine, which failed in a phase II trial in 2009 [43], and ambroxol, whose binding mode is unclear. The iminosugar NN-DNJ was also excluded because of insufficient selectivity [44]. We therefore chose JZ-4109, a quinazoline modulator known to stabilize WT as well as N409S mutant GCase with a clear binding mode [29] (Fig. 1C). As an ATL, we chose ATB1021 [21] (Fig. 1D), which is designed to bind the ZZ domain of p62 and induce its allosteric activation [21]. To synthesize LYSOTAC compounds, JZ-4109 was linked to ATB1021 using a PEG, generating two isoforms, ATB2057 (S-form) and ATB2058 (R-form) with a molecular weight of 972.09 (Fig. 1E). We utilized a standard PEG linker containing heteroatom oxygen to enhance the polarity, flexibility, and biocompatibility of the molecule, as the low-polarity primary alkyl chain can adversely affect physicochemical properties. The reaction of JZ-4109, synthesized as a racemic mixture, with enantiomerically pure ATB1021 produced a mixture of diastereomers, which was subsequently separated by high-performance liquid chromatography (HPLC).

### 3.2 Structural modeling of the LYSOTAC ternary complex

**Fig. 2:**
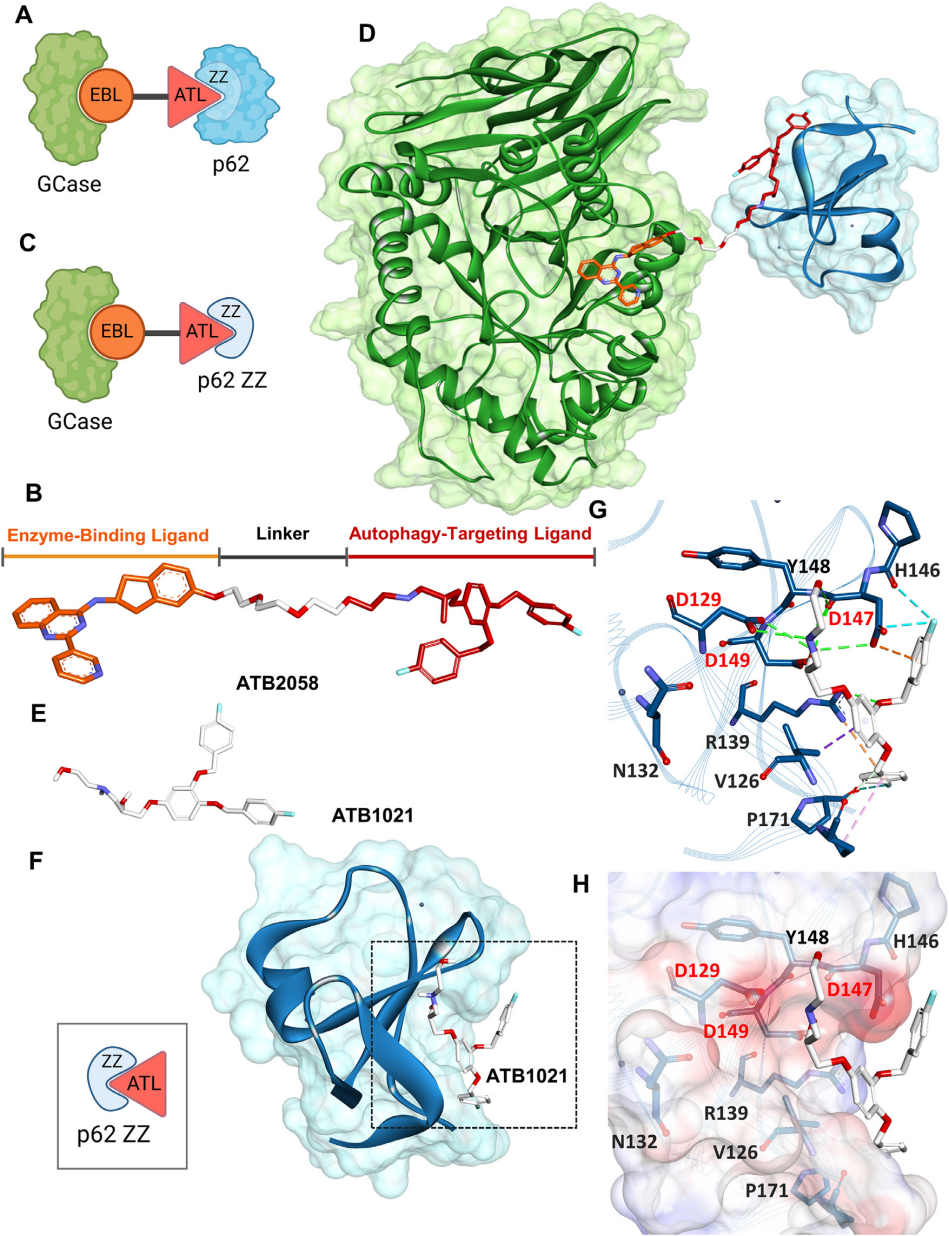
Structural analysis of the LYSOTAC compound binding to GCase and p62 ZZ domain. **(A)** Design of a LYSOTAC compound binding to both GCase and p62 ZZ domain; **(B)** 3D structure of ATB2058 shown as a stick model, consisting of EBL (orange), linker (white), and ATL (red); GCase is shown in green and the p62 ZZ domain in blue, displayed in both cartoon **(C)** and semi-transparent surface representations **(D)**. The LYSOTAC molecule is visualized in the same stick model and color scheme as illustrated in (B); **(E)** 3D structure of the ATL depicted as a white stick model; **(F)** Representative binding conformation of the ATL and p62 ZZ domain (blue) displayed in both cartoon (left panel) and semi-transparent surface representations (right panel); **(G, H)** Interaction analysis of ATB1021 with p62 ZZ domain, with the electrostatic surface. Hydrogen bonds, alkyl, π-alkyl, halogen bonds and electrostatic interactions are represented by dashed lines in green, pink, purple, cyan and orange, respectively. Key interacting residues are colored in red. Cartoons were created in BioRender. Park, J. (2026) https://BioRender.com/6qf7ykt.

ATB2058 was designed to bind both GCase and the p62 ZZ domain (Fig. 2A) in a linear configuration of EBL, linker, and ATL moieties (Fig. 2B). To decipher the molecular principles of LYSOTAC, structural modeling of the ATB2058 ternary complex was conducted using the crystal structure of GCase bound to the EBL JZ-4109 and docking simulations of the ATL ATB1021 with the p62 ZZ domain (Fig. 2C and 2D). Both moieties were positioned within the binding pockets of their respective targets, GCase and p62 ZZ domain (Fig. 2D). As the co-crystal structure of p62 ZZ in complex with ATL has not been defined, a binary complex model of ATB1021 bound to the ZZ domain was also constructed using molecular docking (Fig. 2E and 2F). ATB1021 was positioned within a negatively charged surface pocket of the ZZ domain, with its two fluorophenyl rings inserted into a shallow hydrophobic cavity (Fig. 2F). In the cavity, ATB1021 interacted with key acidic residues in the ZZ domain (D129, D147 and D149) known to recognize and stabilize the Arg/N-degron of physiological substrates through salt bridges and hydrogen bonding (Fig. 2G and 2H) [15,45]. The fluorophenyl rings of ATB1021 formed hydrogen bonds and halogen interactions with these residues, which were further supported by hydrogen bonding with its hydroxyl group at the flexible tail (Fig. 2G and 2H). The EBL-linker moiety conjugated to ATB1021 extended along the solvent-exposed surface of the ZZ domain without occupying the binding pocket. This spatial arrangement allowed the simultaneous binding of both the ATL and EBL with their respective target proteins in the ternary complex, without inducing steric hindrance (Fig. 2D).

In the co-crystal structure of GCase with the EBL, the aromatic rings of the EBL formed π–π and π–alkyl interactions with Y313, L314, F316 and W348 (Fig. S1A). The linker attachment site was located near F316 in a solvent-exposed region, allowing conjugation without disrupting the binding pose (Fig. S1A). The EBL model used in this study corresponded to the R-isomer (ATB2058), and the S-form (ATB2057) was found to adopt a similar conformation within the active site of GCase (Fig. S1B). The binding pocket exhibited sufficient flexibility to accommodate both configurations without steric hindrance or disruption of key interactions (Fig. S1C).

Based on these structural models, the LYSOTAC ternary complex, formed by linking EBL and ATL through a flexible PEG linker, demonstrated simultaneous and stable binding of both target proteins. The overall modeled complex exhibited a well-defined configuration without significant steric clashes, supporting the structural integrity of the LYSOTAC design.

### 3.3 LYSOTAC compounds retain autophagy-targeting activity

**Fig. 3:**
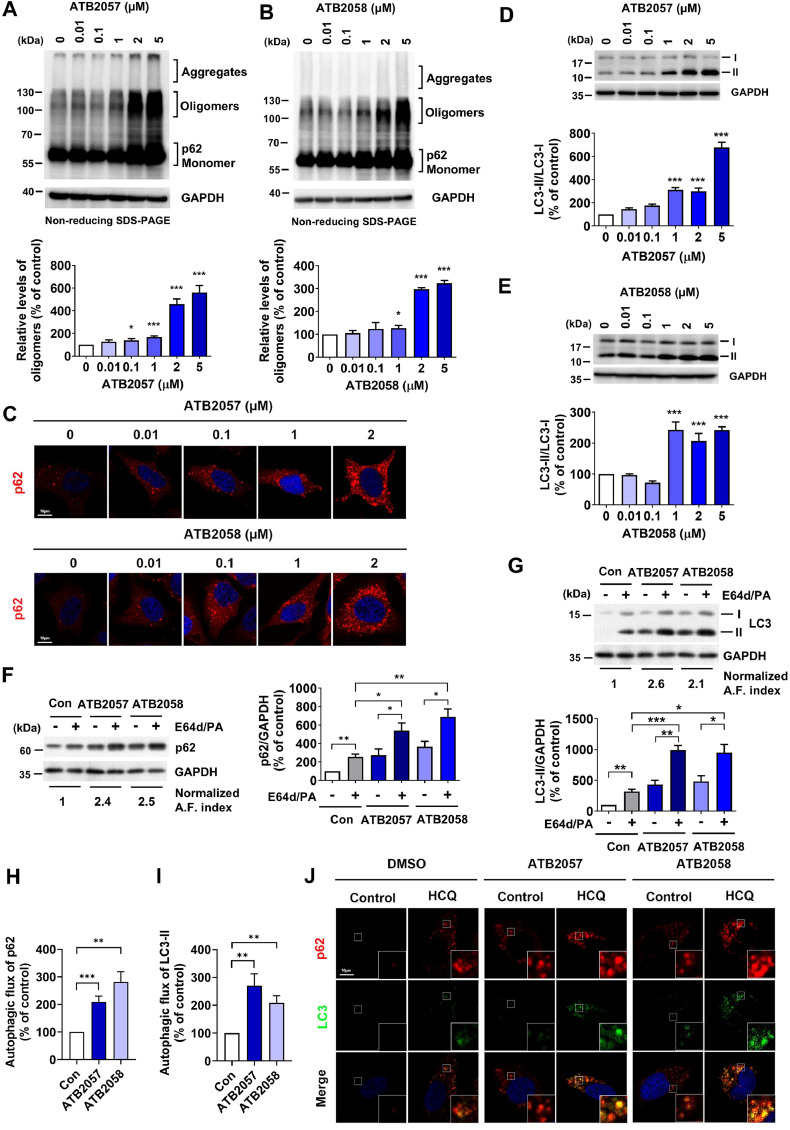
LYSOTAC induces p62 oligomerization leading to the activation of autophagy. WB of HeLa cells treated with **(A)** ATB2057 or **(B)** ATB2058 for 24 h. Quantification of p62 oligomers is displayed below the blots (*n* = 5); **(C)** ICC of p62 puncta in HeLa cells treated with ATB2057 or ATB2058 for 24 h; WB of HeLa cells, treated with **(D)** ATB2057 or **(E)** ATB2058 for 24 h, detecting LC3 (upper panel) and quantification (lower panel, *n* = 5); **(F)** WB of p62 in HeLa cells treated with combination of ATB2057 (2 µM), ATB2058 (2 µM), E64d (10 µg/ml) and pepstatin A (PA, 10 µg/ml) for 24 h (left panel). Quantification of p62/GAPDH is displayed as the right panel (*n* = 5); **(G)** WB of LC3 in HeLa cells treated with combination of ATB2057 (2 µM), ATB2058 (2 µM), E64d (10 µg/ml) and pepstatin A (PepA, 10 µg/ml) for 24 h (upper panel). Quantification of LC3-II/GAPDH is displayed as the lower panel (*n* = 4); **(H)** Autophagic flux calculated by p62 in (F) (*n* = 5); **(I)** Autophagic flux calculated by LC3-II in (G) (*n* = 4). Autophagic flux (A.F.) indices presented beneath the blots in (H) and (I) were calculated as substrate (+ inhibitors) - substrate (- inhibitors). To ensure comparability across experiments, the flux index from each condition was normalized to the control untreated index; **(J)** ICC of p62 and LC3 in HeLa cells exposed to ATB2057 (2 µM, 24 h) or ATB2058 (2 µM, 24 h) in the presence or absence of hydroxychloroquine (HCQ, 25 µM, 18 h). Differences between groups were evaluated by one-way ANOVA. The data are presented as the mean ± SEM. *P < 0.05, **P < 0.01, ***P < 0.001.

The self-oligomerization of p62 in complex with its cargoes is an essential step to form p62-cargo complexes prior to the co-targeting to phagophores [13]. When high-molecular-weight species of p62 linked by disulfide bonds were separated from their monomers using non-reducing sodium dodecyl sulfate-polyacrylamide gel electrophoresis (SDS-PAGE), ATB2057 and ATB2058 exhibited dose-dependent efficacy to induce p62 polymerization (Fig. 3A and 3B). To corroborate these findings, an *in vitro* p62 aggregation assay was performed using extracts from HEK293 cells overexpressing full-length p62. Under conditions in which the total p62 input was held constant, ATB2057 and ATB2058 enhanced aggregate formation, accompanied by a reciprocal decrease in monomeric p62 (Fig. S2A). Because LYSOTAC compounds increased endogenous monomeric p62 in HeLa cells (Fig. 3A and 3B), we additionally assessed p62 expression. The ATL compound ATB1021, as well as the LYSOTAC compounds ATB2057 and ATB2058, increased both *p62* mRNA and protein levels (Fig. S2B and S2C). These results indicate that p62 ligands both induce p62 expression and enhance p62 oligomerization/aggregation. Their lethal concentration 50% (LC_50_) values were estimated to be 2.6 µM, respectively (Fig. S3A). The efficacy to induce p62 polymerization correlated with dose-dependent increases in the formation of p62^+^ cytosolic puncta, as visualized by ICC (Fig. 3C), and LC3 lipidation, as determined by immunoblotting analyses (Fig. 3D and 3E). LYSOTAC compounds conferred the accumulation of both p62 and LC3-II when autophagy flux was blocked using a mixture of E64d and PA that inhibits lysosomal proteases, including cathepsins B, D, E, H and L [46] (Fig. 3F and 3G). The ability of LYSOTAC compounds to induce autophagic flux was confirmed by comparing the levels of p62 and LC3-II in the presence or absence of lysosomal inhibitors (Fig. 3H and 3I). Immunostaining analyses further confirmed that the LYSOTAC compounds stimulated the formation of cytosolic puncta doubly positive for p62 and LC3 (Fig. 3J), indicative of autophagic flux of p62 bodies to LC3^+^ autophagic membranes. These data confirm that the ATB1021 moiety of LYSOTAC compounds exert the activity to target p62 to the lysosome via autophagy.

### 3.4 GCase-LYSOTAC increases levels of post-ER and total GCase

To determine whether GCase-LYSOTAC compounds facilitate the traffic of GCase to the lysosome as an enzymatically active form, we cultured fibroblasts derived from patients with GD carrying *GBA1* G364R/C381G, N409S/L29Afs*18, or L483P/L483P mutations in comparison with fibroblasts from WT individuals (GM08680 and GM05381). Cell viability with ATB2057 or ATB2058 was over 80% up to 2 µM except for N409S/L29Afs*18 cells, which showed 75.6% ± 4.3% and 73.2% ± 1.9% at 1 and 2 µM ATB2058, respectively (Fig. S3B–S3F). Similar to the data from HeLa cells (Fig. 3C), ICC showed that ATB2057 induced p62 puncta formation in fibroblasts in a dose-dependent manner (Fig. S3G).

**Fig. 4:**
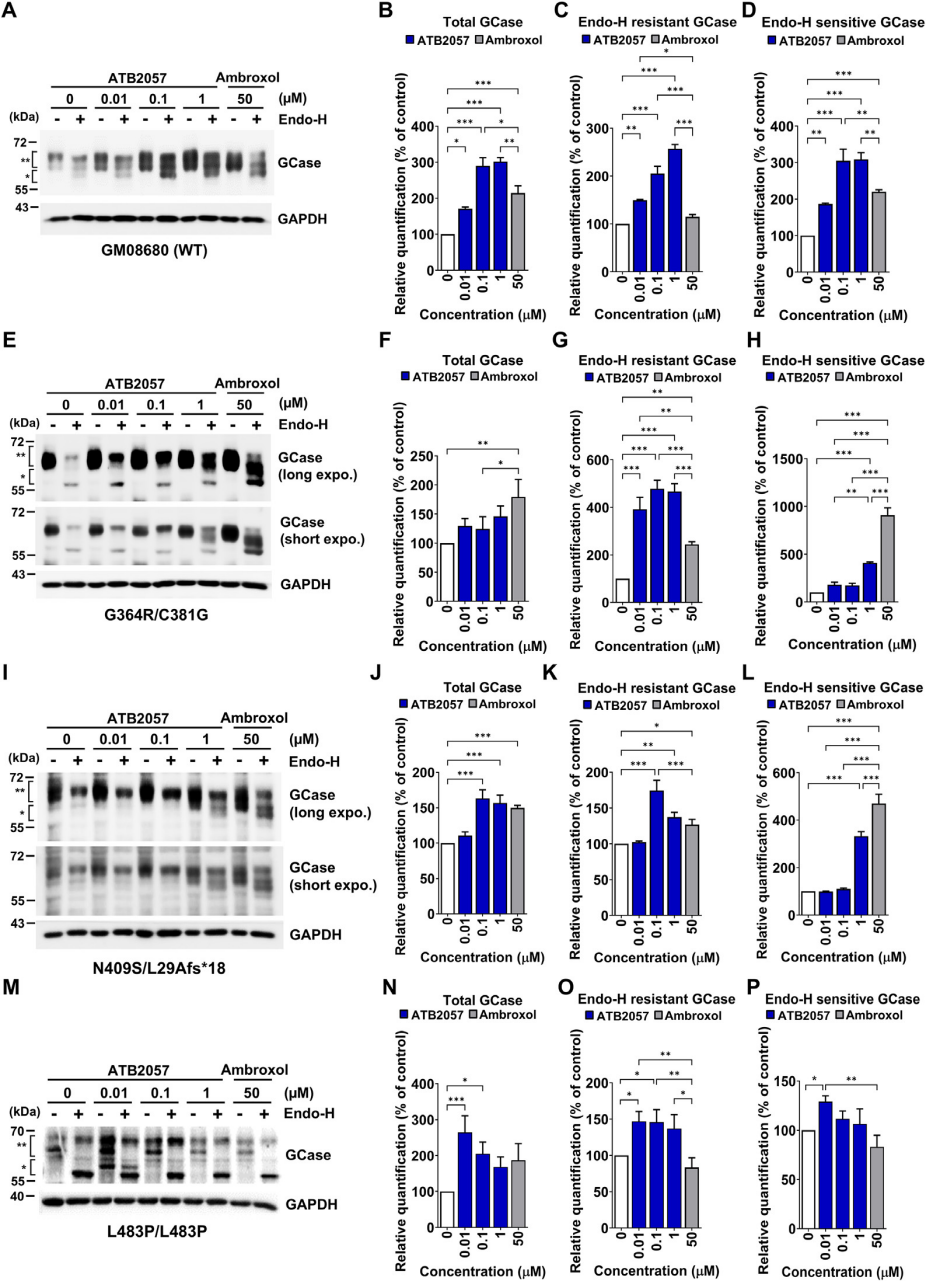
ATB2057 elevates GCase protein levels in normal and GD fibroblasts. Lysates were subjected to Endo-H digestion and the Endo-H resistant and sensitive fractions are marked by ** and *, respectively. **(A)** WB of normal human fibroblasts (GM08680) treated with ATB2057 or ambroxol (50 µM) for 10 d; Quantification of **(B)** total GCase, **(C)** Endo-H resistant GCase and **(D)** Endo-H sensitive GCase of (A) (*n* = 5); **(E)** WB of human GD fibroblasts (GM02627 [G364R/C381G]) treated with ATB2057 or ambroxol (50 µM) for 10 d; Quantification of **(F)** total GCase, **(G)** Endo-H resistant GCase, and **(H)** Endo-H sensitive GCase of (E) (*n* = 5); **(I)** WB of human GD fibroblasts (GM00372 [N409S/L29Afs*18]) treated with ATB2057 or ambroxol (50 µM) for 10 d; Quantification of **(J)** total GCase, **(K)** Endo-H resistant GCase, and **(L)** Endo-H sensitive GCase of (I) (*n* = 5); **(M)** WB of human GD fibroblasts (GM07968 [L483P/L483P]) treated with ATB2057 or ambroxol (50 µM) for 10 d. Quantification of **(N)** total GCase, **(O)** Endo-H resistant GCase, and **(P)** Endo-H sensitive GCase of (M) (*n* = 5). Differences between groups were evaluated by two-way ANOVA. The data are presented as the mean ± SEM. *P < 0.05, **P < 0.01, ***P < 0.001.

Endo-H is a glycosidase enzyme that cleaves mannose-rich N-linked oligosaccharides on premature GCase (ER form) that did not reach the medial Golgi complex, including ER-trapped species [47]. By contrast, mature GCase (post-ER form) is Endo-H-resistant (Endo-H^R^). Immunoblotting analyses showed that both ATB2057 and ATB2058 markedly elevated the levels of total as well as ER and post-ER GCase in GM08680 and GM05381 WT fibroblasts (Figs. 4A–4D, S3H–S3K and S4A–S4H). Similarly, ATB2057 and ATB2058 significantly restored the levels of total, ER and post-ER GCase in N409S/L29Afs*18, G364R/C381G and L483P/L483P GD fibroblasts (Figs. 4E–4P and S4A–S4L). Importantly, ATB2057 was more effective than ambroxol in preserving the post-ER forms of GCase in both normal and GD fibroblasts (Figs. 4C, 4G, 4K, 4O and S3J). The elevation of Endo-H^R^ GCase levels by ATB2057 and ATB2058 exhibited a dose-dependent pattern in normal fibroblasts (Figs. 4C, S3J, S4C and S4G). The maximal effects varied depending on the origin of fibroblasts (1 µM in normal fibroblasts and 0.1 µM in GD fibroblasts) (Figs. 4C, 4G, 4K, 4O, S3J, S4C, S4G, S5C, S5G and S5K), possibly due to the difference in cytotoxicity of LYSOTAC compounds in each fibroblast (Fig. S3B–S3F) and the mutation properties. Based on these observations, 0.1 µM was selected as the primary treatment concentration for fibroblasts. Considering both cytotoxicity (Fig. S3B–S3D) and the capacity to restore post-ER forms of GCase (Figs. 4 and S5), ATB2057 exhibited overall superior efficacy compared with ATB2058.

Notably, the recovery of GCase abundance was mostly superior in ATB2057-treated groups than in ambroxol-treated groups. In comparison with ambroxol, Endo-H^R^ post-ER forms were more efficiently restored in LYSOTAC-implemented GD fibroblasts. It is known that GCase, located in the ER or in early regions of the Golgi complex, remains sensitive to Endo-H, whereas it acquires Endo-H resistance after being processed by the enzymes located in the medial-Golgi complex [48]. Therefore, the increase in Endo-H^R^ GCase levels by LYSOTAC treatment suggests that GCase is processed through the medial-Golgi apparatus, which is essential for the recovery of catalytic activity. This could be achieved by either the chaperone effect of JZ-4109 or by organelle-specific autophagy, such as Golgi-phagy via p62 activation. It remains to be investigated whether LYSOTAC-engaged p62 directly facilitates the transportation of GCase from the Golgi apparatus to the lysosome.

### 3.5 GCase-LYSOTAC recovers GCase enzymatic activity in GD fibroblasts

**Fig. 5:**
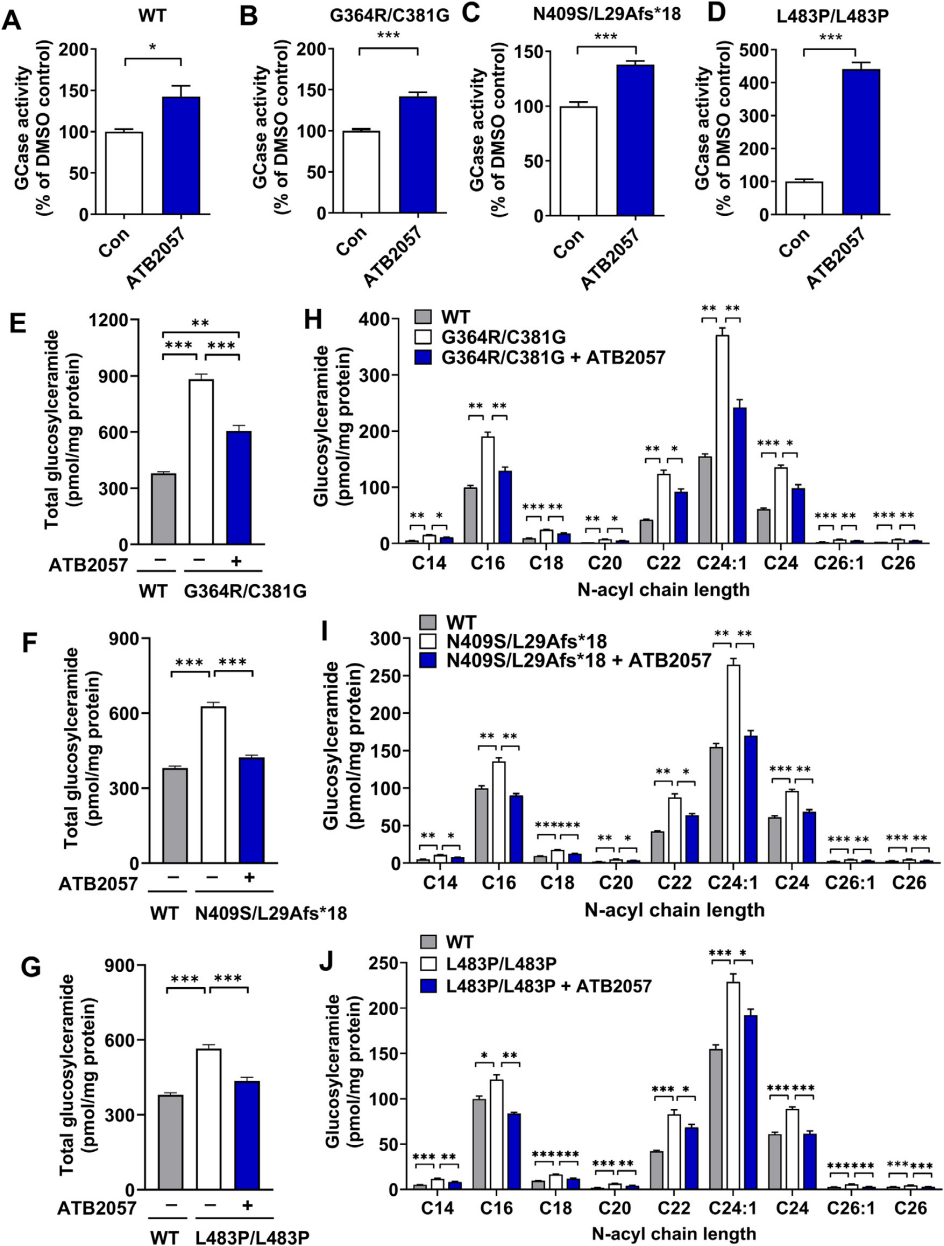
GCase activity is increased by ATB2057 in GD fibroblasts, leading to the reduction of glucosylceramide. GCase activity in **(A)** WT (GM08680) and GD fibroblasts [**(B)** GM02627, **(C)** GM00372 and **(D)** GM07968] treated with ATB2057 (0.1 µM) for 10 d. Data are displayed as percentage of DMSO-treated control (*n* = 5); Total glucosylceramide levels in WT and GD fibroblasts [**(E)** GM02627, **(F)** GM00372 and **(G)** GM07968] with or without ATB2057 (0.1 µM, 10 d) treatment (*n* = 3); Acyl chain length of glucosylceramide in GD fibroblasts [**(H)** GM02627, **(I)** GM00372, and **(J)** GM07968)] following treatment of ATB2057 (0.1 µM, 10 d (*n* = 3). Differences between groups were evaluated by unpaired Student’s *t*-test (A-D), one-way ANOVA (E-G), or two-way ANOVA (H-J). The data are presented as the mean ± SEM. *P < 0.05, ***P < 0.001.

To assess the therapeutic efficacy of LYSOTAC compounds, the enzymatic activity of GCase was examined in WT and GD fibroblasts. The GCase activity of WT fibroblasts (GM08680) was 247.3 ± 7.5 nmol/h/mg, which increased to 352.6 ± 32.2 nmol/h/mg when treated with 0.1 µM ATB2057 for 10 d (Fig. S6A). The basal GCase activities in G364R/C381G and N409S/L29Afs*18 GD cells were 38.9 ± 1.0 and 29.4 ± 1.3 nmol/h/mg, respectively, which increased to 55.2 ± 1.9 and 40.5 ± 1.0 nmol/h/mg upon ATB2057 treatment (Fig. S6B and S6C). L483P/L483P GD cells showed the most severe GCase deficiency with the basal activity of 2.5 ± 0.2 nmol/h/mg, which improved to 10.8 ± 0.5 nmol/h/mg (Fig. S6D). The percentage increase of GCase activity by ATB2057 was 142.6% ± 13.0% in WT fibroblasts and 142.1% ± 15.2%, 137.9% ± 10.9% and 440.6% ± 65.5% in G364R/C381G, N409S/L29Afs*18 and L483P/L483P cells, respectively (Fig. 5A–5D).

JZ-4109, the EBL moiety in AUTOTAC compounds, functions as a pharmacological chaperone by stabilizing misfolded GCase, despite its intrinsic inhibitory activity toward the enzyme [29]. Upon reaching the acidic lysosomal environment, JZ-4109 dissociates from GCase, contributing to restoring the residual enzyme activity [49]. Such efficacy of JZ-4109 was shown at 2 µM for GCase trafficking to the lysosome in HEK293 cells, as well as the levels of post-ER GCase and its enzymatic activity in WT and N409S GD fibroblasts [29]. To rule out the possibility that the efficacy of LYSOTAC compounds on GCase stems from their EBL or ATL, we conducted analogous assays with JZ-4109 and ATB1021, following the treatment at 0.1 µM for 10 d. Although GCase activity increased slightly with JZ-4109 in G364R/C381G cells, overall, EBL or ATL alone exhibited no significant efficacy (Fig. S6E–S6L). Importantly, 0.1 µM ATB2057 was sufficient to elevate GCase activity compared with JZ-4109 or ATB1021 alone (Fig. S6M–S6T). Given the modest increase in GCase activity observed with JZ-4109 in G364R/C381G cells (Fig. S6F), we prepared lysosome-enriched fractions to evaluate the efficiency of lysosomal delivery of ATB2057 compared with JZ-4109 alone. In these analyses, ATB2057 increased lysosomal delivery of GCase in GD fibroblasts compared with JZ-4109 alone (Fig. S7A and S7B), highlighting its superior potency and potential therapeutic advantage.

During the pathogenesis of GD, GCase deficiency leads to excessive accumulation of its substrate, glucosylceramide. The concentration (pmol/mg protein) of glucosylceramide was 379.9 ± 8.3 in WT cells (Fig. 5E). In G364R/C381G GD cells, it was significantly elevated to 882.2 ± 26.8, which was reduced to 605.4 ± 29.5 by ATB2057 treatment (Fig. 5E). A similar efficacy of ATB2057 was reproduced in N409S/L29Afs*18 (from 627.7 ± 15.7 to 423.2 ± 8.9) and L483P/L483P (from 566.2 ± 15.0 to 435.7 ± 15.1) (Fig. 5F and 5G). The reduction in the levels of glucosylceramide species was invariably observed in all GD fibroblasts regardless of their acyl chain lengths (Fig. 5H–5J). These data indicate the therapeutic efficacy of LYSOTAC to lower the pathogenic toxic substance in GD.

### 3.6 GCase-LYSOTAC facilitates trafficking of GCase to lysosomes

**Fig. 6:**
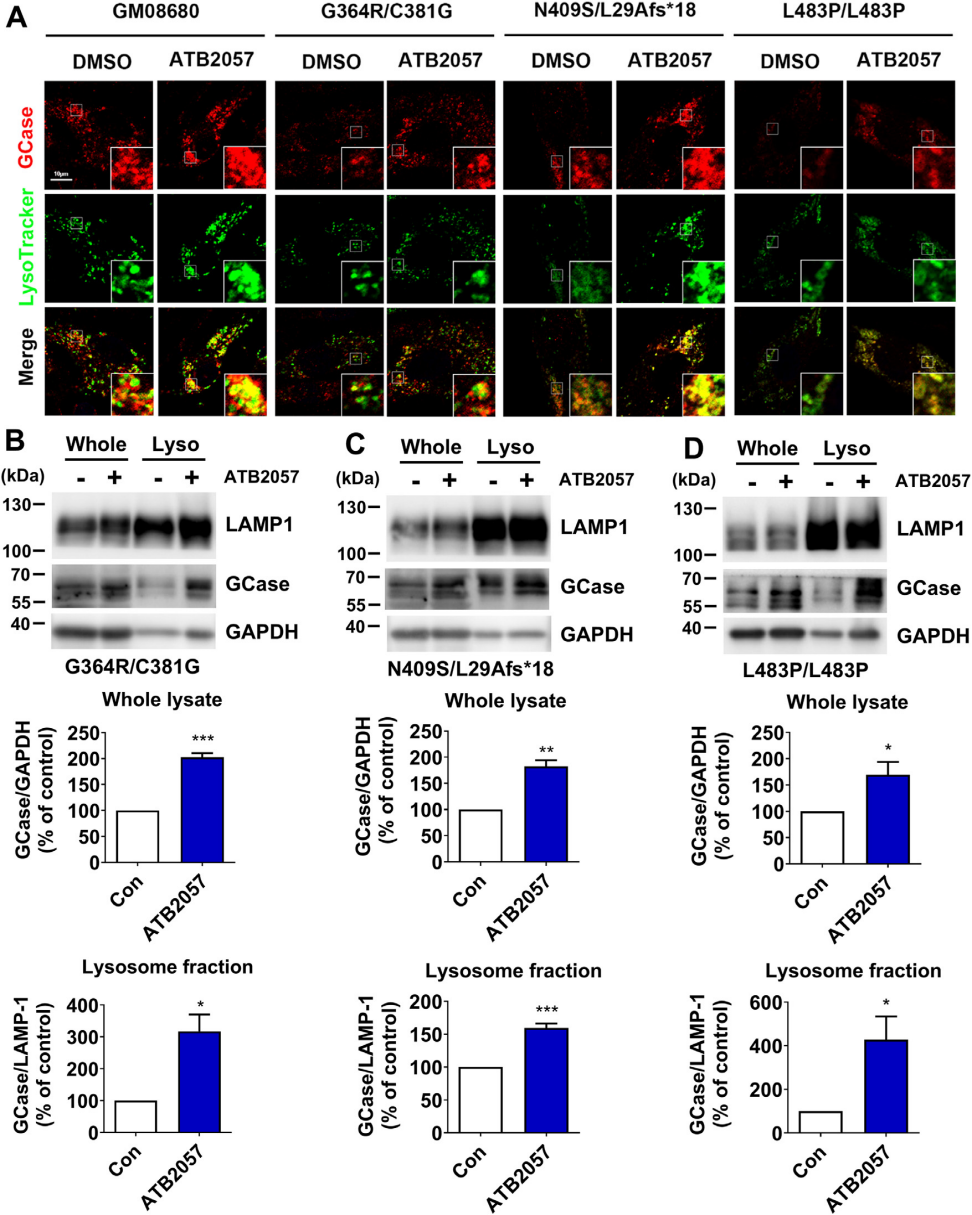
Lysosomal delivery of GCase is enhanced by ATB2057. **(A)** ICC of GCase (Alexa Fluor 647) with LysoTracker staining (DND-99, pseudo-colored green) in fibroblasts incubated with or without ATB2057 (0.1 µM, 2 d); Following treatment with ATB2057 (0.1 µM, 2 d), GD fibroblasts [**(B)** GM02627, **(C)** GM00372, and **(D)** GM07968)] were fractionated. Whole cell lysates (Whole) in comparison with lysosome (Lyso) fractions were analyzed by WB. Quantification of GCase is shown below the blots (*n* = 5). Differences between groups were evaluated by unpaired Student’s *t*-test. The data are presented as the mean ± SEM. *P < 0.05, **P < 0.01, ***P < 0.001.

We determined whether GCase-LYSOTAC facilitates the traffic of GCase to the lysosome using ICC. In WT GM08680 fibroblasts, the treatment of 0.1 µM ATB2057 significantly increased the colocalization of GCase signals with those of LysoTracker, which exhibits a pH-dependent increase in fluorescence intensity upon acidification (Fig. 6A). The lysosomal targeting of GCase was similarly observed in G364R/C381G, L483P/L483P and N409S/L29Afs*18 GD fibroblasts (Fig. 6A). By contrast, no significant changes were observed in the lysosomal-associated membrane protein 1 (LAMP1) levels (Fig. S8A–S8E) and lysosomal biogenesis–related genes (*LAMP1, M6PR* and *MCOLN1*) (Fig. S8F–S8N), suggesting that ATB2057 did not promote lysosomal biogenesis. When GD fibroblasts treated with ATB2057 were fractionated and subjected to immunoblotting analyses, the enhanced total level of GCase correlated with its increased lysosomal localization (Fig. 6B–6D). These data demonstrate that ATB2057 confers the targeted delivery of GCase to the lysosome.

### 3.7 LYSOTAC overcomes LIMP-2 deficiency and enhances GCase activity as well as its protein level

**Fig. 7:**
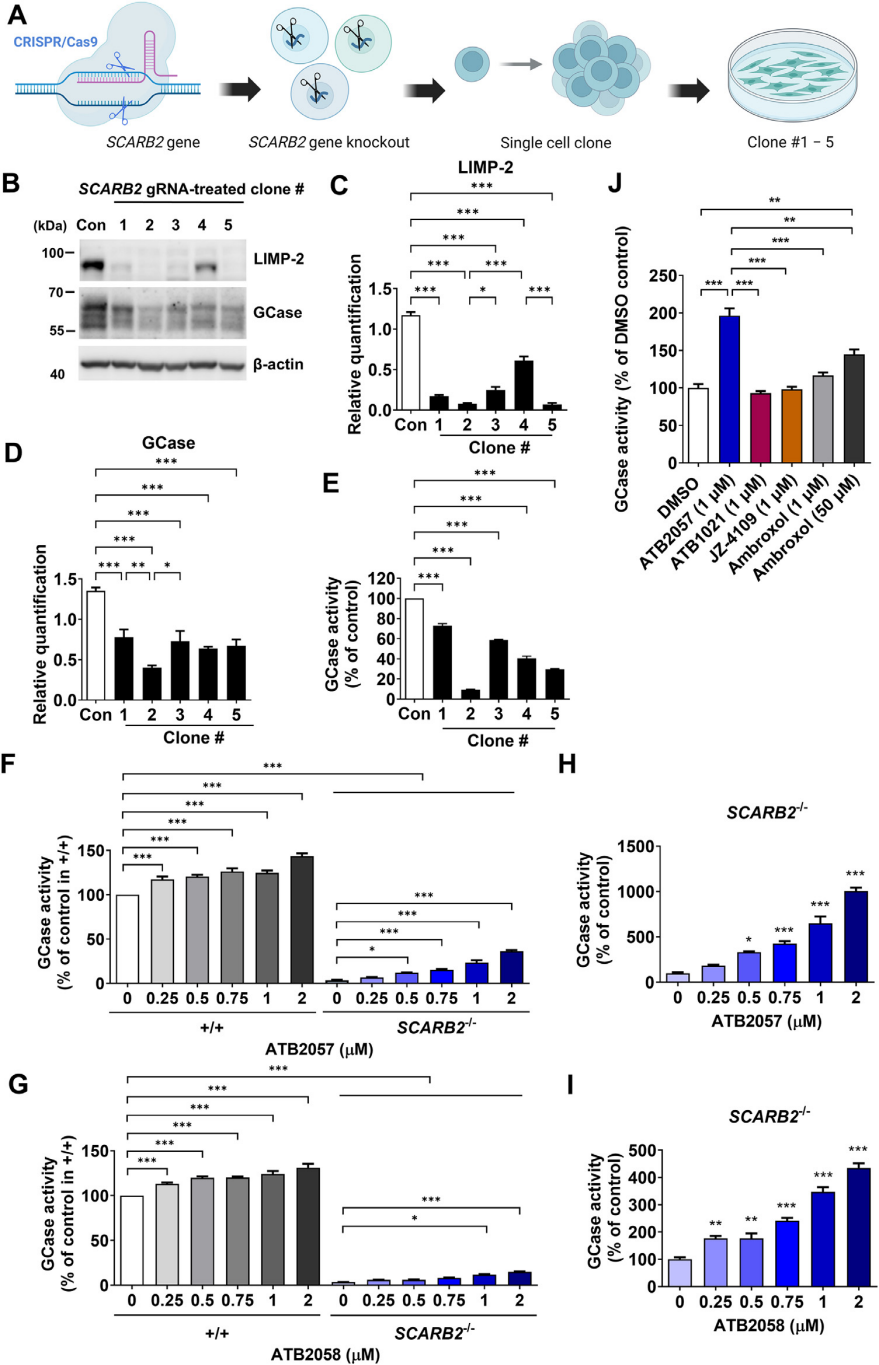
LYSOTAC partially recovers GCase activity in LIMP-2 deficient HEK293 cells. **(A)** Scheme of generating *SCARB2*^-/-^ clones (Created in BioRender. Park, J. (2026) https://BioRender.com/6qf7ykt); **(B)** WB of HEK293 cell clones transfected with gRNA vector targeting *SCARB2*; Relative quantification of **(C)** LIMP-2 and **(D)** GCase compared to control HEK293 cells in (B) (*n* = 5); **(E)** GCase activity of HEK293 cell clones transfected with gRNA vector targeting *SCARB2* (*n* = 5); GCase activity of LIMP-2 deficient HEK293 cells (clone #2) exposed to **(F)** ATB2057 or **(G)** ATB2058 for 7 d was compared with control HEK293 cells (*n* = 5); **(H, I)** Increased GCase activity of LIMP-2 deficient HEK293 cells in (F) and (G) is shown as a percentage change (*n* = 5); **(J)** GCase activity of LIMP-2 deficient HEK293 cells treated with ATB2057 (1 µM), ATB1021 (1 µM), JZ-4109 (1 µM), and ambroxol (1 or 50 µM) for 7 d. Differences between groups were evaluated by one-way ANOVA (C, D, E, H, I, and J) or two-way ANOVA (F and G). The data are presented as the mean ± SEM. *P < 0.05, **P < 0.01, ***P < 0.001.

Unlike other lysosomal enzymes targeted via mannose 6-phosphate (Fig. 1A), GCase is transported to the lysosome by binding to LIMP-2 [50]. GCase binds to LIMP-2 in the ER and remains bound to LIMP-2 during trafficking to the Golgi, the *trans*-Golgi network, and the endosomal compartment [50]. GCase is delivered to lysosomes after the acidic pH in the late endosomal compartment liberates GCase from LIMP-2 [50]. We therefore investigated whether p62-mediated autophagy can deliver GCase to the lysosome independently of LIMP-2, which is encoded by *SCARB2*. To this end, we generated LIMP-2-deficient HEK293 cells using Clustered Regularly Interspaced Short Palindromic Repeats (CRISPR)/CRISPR-associated protein 9 (Cas9) technology (Fig. 7A). *SCARB2*-gRNA-treated clones displayed a reduction in the abundance of not only LIMP-2 but also GCase (Fig. 7B), confirming a role of LIMP-2 as the trafficking receptor of GCase to the lysosome. Among the five clones generated, LIMP-2 was hardly detected in clones #2 and #5 (Fig. 7C). We further characterized clone #2, in which the level of GCase and its enzymatic activity were diminished to the greatest degree (Fig. 7D and 7E). In control HEK293 cells, the treatment with ATB2057 or ATB2058 for 7 d led to a dose-dependent elevation of GCase activity, with maximal increases of 143.4% ± 3.2% and 131.2% ± 4.2% at 2 µM, respectively (Fig. 7F and 7G). Importantly, in LIMP-2-deficient cells treated with 2 µM ATB2057 and ATB2058 for 7 d, GCase activity was increased by 1005.6% ± 376.8% and 435.3% ± 17.1%, respectively (Fig. 7H and 7I). Given that the basal GCase activity of LIMP-2-deficient HEK293 cells was 3.5% ± 0.2% of DMSO-treated WT controls, GCase-LYSOTAC compounds restored the GCase activity in LIMP-2-deficient cells to 36.2% ± 1.4% and 14.8% ± 0.6% of normal levels (Fig. 7F and 7G). ATB2057 exerted superior efficacy not only over ATL and EBL when treated at 1 µM for 7 d but also over ambroxol treated at 50 µM for 7 d (Fig. 7J). These results, in line with the GCase activity data of fibroblasts (Fig. 5A–5D), further underscore the enhanced potency of ATB2057 relative to JZ-4109.

To investigate which form of GCase was restored by LYSOTAC, we also conducted Endo-H digestion assays followed by immunoblotting analyses. Whereas ATB2057 and ATB2058 increased the total, ER and post-ER forms of GCase in WT cells (Fig. S9A and S9B), such increases were observed with only the total and ER form of GCase in LIMP-2-deficient cells (Fig. S9C and S9D). These results suggest that LIMP-2 is essential to produce the post-ER GCase form (Fig. S10A and S10B).

Our results show that both ATB2057 and ATB2058 significantly restore GCase levels in LIMP-2-deficient cells, demonstrating the lysosomal delivery efficacy of LYSOTAC compounds irrespective of LIMP-2. The GCase species rescued by LYSOTAC compounds were mostly Endo-H-sensitive ER form, reaffirming the role of LIMP-2 in GCase transportation through the Golgi, in which Endo-H resistance is acquired, to the endosomal compartments [50]. Nevertheless, LYSOTAC-induced enhancement of GCase delivery to the lysosome partially recovered GCase activity in LIMP-2-deficient cells. Due to cell-type-specific differences in basal GCase activity and susceptibility to LYSOTAC compound-induced cytotoxicity (Figs. S3A–S3F, S11A–S11B), compound concentrations were adjusted for each cell type to ensure a balance between therapeutic efficacy and cellular viability.

### 3.8 GCase-LYSOTAC promotes lysosomal transportation of GCase via p62

We examined whether p62-mediated autophagy is involved in the lysosomal targeting of GCase. siRNA-mediated *p62* knockdown in GD fibroblasts partially abrogated ATB2057-induced restoration of GCase protein levels (Fig. S12A–S12C). Additionally, increased colocalization of GCase with LysoTracker by ATB2057 was no longer detected in *p62*^-/-^ cells, in contrast to the WT control (Fig. S12D). These data confirm that the mode of action of LYSOTAC is mediated through p62.

## 4 Conclusions

Autophagy is generally considered a conserved intracellular process in which intracellular cargo materials are delivered to lysosomes for degradation and recycling. Based on the fact that the final destination of autophagic cargoes is the lysosome, we hypothesized that autophagic flux could be utilized to deliver LSD-associated enzymes to their proper destination. To test this hypothesis, we developed the LYSOTAC technology, which employs a chimeric compound consisting of the EBL linked to the ATL that binds and activates p62. ATB2057 and ATB2058 isomers adopted JZ-4109 as an EBL because its binding mode and chaperone effect for GCase have been well established [29]. JZ-4109 was conjugated to a PEG linker, which in turn was conjugated to ATB1021 [25] as an ATL that binds and activates p62. ATB2057 and ATB2058 effectively accelerated lysosomal trafficking of GCase with concomitant increases in the level and enzymatic activity of GCase in a manner dependent on p62-mediated autophagy. These increases correlated with a significant restoration of the post-ER form of GCase, resulting in a reduced abundance of cytotoxic glucosylceramide in GD fibroblasts. Both ATB2057 and ATB2058 exhibited *in vitro* therapeutic efficacy at nanomolar ranges in GD fibroblasts. The efficacy of these prototype compounds was superior to that of ambroxol, which has been evaluated in several Phase 2 clinical trials for GD [51,52].

In ERT, mannose residues on *N*-glycans of recombinant GCase are engineered to target and facilitate internalization into macrophages [53]. Other lysosome-targeted therapeutic enzymes undergo the modification of mannose 6-phosphate in the *N*-glycans, which generates an address tag for the recognition and trafficking of lysosomal enzymes [54]. These manufacturing processes on a large scale are very complex, sophisticated, and time-consuming, making the drug very expensive. Moreover, the current ERT strategy is a lifelong treatment that often requires regular, sometimes weekly, intravenous infusions, with annual costs up to several hundred thousand dollars. In contrary, the lysosomal delivery system based on p62-mediated autophagy is simple and direct, which does not demand N-glycan processing. Furthermore, chemical based LYSOTAC system would not generate neutralizing antibodies, which adversely affect therapeutic efficacy of ERT.

GD is typically diagnosed by measuring the deficient activity of GCase in peripheral blood leukocytes. A diagnosis of GD is confirmed when enzymatic activity is <15% of the standard activity observed in healthy individuals. As the current study was conducted in fibroblasts rather than leukocytes, precise therapeutic efficacy cannot be directly extrapolated. Nevertheless, LYSOTAC treatment in GD fibroblasts (N409S/L29Afs*18 and L483P/L483P) significantly reduced glucosylceramide levels comparable with WT fibroblasts. Given that in LSDs, an enzyme activity of approximately 20% of normal is generally sufficient for clinical benefit [55], the partial restoration of GCase activity conferred by LYSOTAC may be sufficient to exert therapeutic efficacy. A more definitive conclusion about the therapeutic potential of LYSOTAC will require *in vivo* experiments, for example, using mice carrying *Gba1* point mutations like *Gba1*^V394L/V394^^L^ and *Gba1*^D409V/D409V^. It is therefore necessary to characterize the pharmacokinetic (PK) profiles of LYSOTAC compounds. In a previous study [26], AUTOTAC ATC161, which adopted a PEG linker conjugated to an ATL with structural similarity to ATB1021, exhibited excellent PK profiles in mice. For example, oral administration of ATC161 at 10 mg/kg resulted in systemic exposure of 844 ng·h/ml (AUClast) with a bioavailability of 21%, a half-life (T_1/2_) of 5.3 h, and a brain-to-plasma ratio of up to 10% at 5 mg/kg. In investigational new drug (IND)-enabling Good Laboratory Practice (GLP) studies, the no-observed-adverse-effect-level (NOAEL) values were at least 240 and 500 mg/kg in rats and beagle dogs, respectively, upon oral administration 3 times/week for 4 weeks (data not shown). ATC161 is under Phase 1 clinical trials at Seoul National University Hospital in South Korea with 76 healthy volunteers (202300697), aiming to treat Alzheimer's disease and progressive supranuclear palsy. Given the structural similarity of LYSOTAC compounds to ATC161 in the linker-ATL moiety, they are likely to exhibit similar PK profiles, including BBB penetration. If so, intravenous injection at 10 mg/kg will be sufficient to expose ∼5 µM in the blood to treat type 1 GD and ∼600 nM in the brain to treat type 2 or 3 GD. Importantly, both ∼5 µM in the blood and ∼600 nM in the brain are significantly higher than 100 nM, which was sufficient to exert therapeutic efficacy *in vitro*. Further structural optimization may be required to reduce the moderate cytotoxicity observed at 2 µM for potential preclinical and clinical applications. We suggest that optimization of ATB2057 may provide a novel therapeutic strategy to treat all types of GD.

## CRediT authorship contribution statement

**Hee-Yeon Kim:** Writing – original draft, Validation, Investigation, Formal analysis, Data curation. **Eun Nam Choi:** Project administration, Investigation, Formal analysis, Data curation. **Gee Eun Lee:** Writing – review & editing, Resources, Project administration, Methodology. **Sanghwa Yoon:** Validation, Methodology, Investigation, Formal analysis, Data curation. **Su Ran Mun:** Investigation, Formal analysis, Data curation. **Eui Jung Jung:** Project administration, Methodology. **Minji Kim:** Methodology, Investigation. **Hyomin Lim:** Methodology, Investigation. **Yang Jae Kang:** Supervision, Software, Investigation, Formal analysis, Data curation. **Woo-Jae Park:** Writing – review & editing, Supervision, Investigation, Formal analysis, Data curation. **Yong Tae Kwon:** Writing – review & editing, Supervision, Conceptualization. **Joo-Won Park:** Writing – review & editing, Writing – original draft, Supervision, Investigation, Funding acquisition, Data curation, Conceptualization.

## Conflicts of interest

Seoul National University and Ewha Womans University have filed patent applications based on the results of this study.

## Appendix Supplementary materials

Image, application 1
