# Supplementary material for: Targeted delivery of glucocerebrosidase to lysosomes: The LYSOTAC (LYSOsome-TArgeting Chimera) technology
Source: Asian J Pharm Sci. 2026 Mar 16;21(2):101149. doi: 10.1016/j.ajps.2026.101149 (PMC13099342; doi:10.1016/j.ajps.2026.101149)
Supplement: Supplementary file 1 [file mmc1.docx]

**Supplemental Materials**

**Targeted delivery of glucocerebrosidase to lysosomes: the LYSOTAC (LYSOsome-TArgeting Chimera) technology**

Hee-Yeon Kim^1, #^, Eun Nam Choi^1, #^, Gee Eun Lee^2^, Sanghwa Yoon^3^, Su Ran Mun^2^, Eui Jung Jung^2, 4^, Minji Kim^1^, Hyomin Lim^1^, [Yang Jae Kang](https://pubmed.ncbi.nlm.nih.gov/?sort=date&term=Kang+YJ&cauthor_id=39719584)^3, 5, 6^, Woo-Jae Park^7, *^, Yong Tae Kwon^2, 8, 9, 10, 11, *^, and Joo-Won Park^1, *^

^1^ Department of Biochemistry, College of Medicine, Ewha Womans University, Seoul 07804, Republic of Korea

^2^ Cellular Degradation Biology Center and Department of Biomedical Sciences, College of Medicine, Seoul National University, Seoul 03080, Republic of Korea.

^3^ Research Institute of Molecular Alchemy, Gyeongsang National University, Jinju 52828, Republic of Korea

^4^ Department of Medicine, Columbia University Irving Medical Center, New York, NY 10032, USA

^5^ Division of Bio & Medical Bigdata Department (Brain Korea 21 Four), Gyeongsang National University, Jinju 52828, Republic of Korea

^6^ Division of Life Science Department, Gyeongsang National University, Jinju 52828, Republic of Korea

^7^ Department of Biochemistry, Chung-Ang University College of Medicine, Seoul 06974, Republic of Korea

^8^ AUTOTAC Bio Inc., 225 Gasan digital 1-ro, Geumcheon-gu, Seoul 08501, Republic of Korea

^9^ Ischemic/Hypoxic Disease Institute, College of Medicine, Seoul National University, Seoul 03080, Republic of Korea

^10^ Convergence Dementia Research Center, Seoul National University Medical Research Center, Seoul 03080, Republic of Korea

^11^ National Research Laboratory for Convergence Degradation Biology, Korea University, Seoul 02841, Republic of Korea

**Supplementary Materials and Methods**

**Chemical synthesis and analytic data of LYSOTACs**

^1^H NMR spectra was recorded on Bruker Avance III 400 MHz and Bruker Fourier 200 MHz with TMS as an internal standard.

LC-MS was taken on a quadrupole Mass Spectrometer on Agilent 1260HPLC and 6120MSD (Column: C18 (50 × 4.6 mm, 5 μm) operating in ES (+) or (-) ionization mode; T = 30 ^o^C; flow rate = 1.5 mL/min; detected wavelength: 210 nm.

**Scheme 1.** Synthesis of compound 2

A solution of compound 1 (20.0 g, 0.12 mol, 1.0 eq) in MeOH (300 mL) was heated at 45 °C,

n-butyl nitrile (16.0 mL) was added to the mixture, stirred for 0.5 h, then HCI (12N, 12 mL)

was added slowly. The mixture was stirred at this temperature for 1 h, then cooled to room

temperature and filtered. The filter cake was washed with MTBE and dried in vacuum to give compound 2 (20.0 g, 84.7%) as white solid. (TLC: PE/EA = 10/1, R_f_ = 0.6)

^1^H NMR (DMSO_d6, 40 MHz): δ 12.48 (s, 1H), 7.69 (d, J = 8.8 Hz, 1H), 7.15 (s, 1H),

7.01-7.03 (m, 1H), 3.89 (s, 3H), 3.73 (s, 2H).

**Scheme 2.** Synthesis of compound 3

To a mixture of compound 2 (20.0 g, 0.10 mol, 1.0 eq) in AcOH (300 mL) was added conc.

H_2_SO_4_ (25 mL), followed by Pd/C (10%, 10 g). The suspension was degassed and flushed with H_2_ for three times, then bubbled with H_2_ to keep the pressure in 50 psi, and stirred at 50 °C overnight. The suspension was cooled to room temperature and filtered. The filtrate was concentrated in vacuum to remove most AcOH. The residue was poured into ice-water, and adjusted pH to 10 with aq. NaOH (2 N), then extracted with EA (200 mL x 3). The combined organic layers were washed with brine, dried over Na_2_SO_4_ and concentrated in vacuum to remove most EA. To the residue was dropwise HCI/EA (5 M, 30 mL), stirred for 0.5 h, filtered. The filter cake was dried in vacuum to give the HCl salt of compound 3 (12.0 g, crude) as white solid. (TLC: DCM/MeOH = 20/1, R_f_ = 0.6)

^1^H NMR (DMSO_d6, 400 MHz): δ 8.44 (brs, 2H), 7.13-7.16 (m, 1H), 6.86 (s, 1H), 6.74-6.76 (m, 1H), 3.94-3.98 (m, 1H), 3.71 (s, 3H), 3.10-3.25 (m, 2H), 2.88-3.01 (m, 2H).

**Scheme 3.** Synthesis of compound 5

A mixture of compound 4 (10.0 g, 1.0 eq) and nicotinoyl chloride hydrochloride (19.6 g, 110.1 mmol, 1.3 eq) in sulfolane (100 ml) was heated to 100 °C and stirred for 4 h. The mixture was cooled to the room temperature and poured into water and extracted with EA (200 mL x 3). The combined organic layers were washed with water, brine, dried over Na_2_SO_4_ and concentrated in vacuum. The crude product purified by silica gel, eluted with PE/EA (10:1~3:1) to give the compound 5 (6.20 g, 33.9%) as yellow solid. (TLC: DCM/MEOH = 20/1, R_f_ = 0.4)

^1^H NMR (DMSO_d6, 400 MHz): δ 10.88 (s, 1H), 9.18 (s, 1H), 8.83 (s, 1H), 8.34-8.36 (m, 1H), 7.90-7.92 (m, 1H), 7.78-7.80 (m, 1H), 7.61-7.64 (m, 2H), 7.45-7.49 (m, 1H).

**Scheme 4.** Synthesis of compound 6

To a mixture compound 5 (6.40 g, 28.7 mmol, 1.0 eq) in sulfolane (65mL) was added PCL_5_ (18.0 g, 86.1 mmol, 3.0 eq) at 100 °C. The mixture was stirred at 100 °C for 10 h. The mixture was poured into water and then adjusted pH to 10 with Na_2_CO_3,_ filtered. The filer cake was dried in vacuum to give compound 6 (5.50g, 79.3%) as yellow solid. (TLC: DCM/MeOH = 20/1, R_f_ = 0.6)

^1^H NMR (CDCl_3_, 400 MHz): δ 9.81 (s, 1H), 8.85-8.88 (m, 1H), 8.78 (s, 1H), 8.31 (dd, J = 0.8, 8.4 Hz, 1H), 8.41 (d, J = 8.4 Hz, 1H), 7.98-8.04 (m, 1H), 7.72-7.76 (m, 1H), 7.47-7.50 (m, 1H).

**Scheme 5.** Synthesis of compound 7

To a mixture of compound 6 (5.50 g, 22.7 mmol, 1.0 eq) in DMF (55 mL) was added compound 6 hydrochloride (4.50 g, 22.7 mmol, 1.0 eq) and K_2_CO_3_ (6.30 g, 45.5 mmol, 2.0 eq). The mixture was heated to 60 °C and stirred for 3 h. The mixture was cooled to room temperature and poured into water and extracted with EA (150 mL x 3). The combined organic phases were washed with water, brine, dried over Na_2_SO_4_ and concentrated in vacuum. The residue was purified by silica gel, eluted with PE/EA (3:1~1:1) to give compound 7 (3.00 g, 35.8%) as white solid. (TLC: DCM/MEOH = 20/1, R_f_ = 0.4)

^1^H NMR (CDCl_3_, 400 MHz): δ 9.81 (s, 1H), 8.91 (d, J = 8.0 Hz, 1H), 8.73 (s, 1H), 7.99-8.02 (m, 1H), 7.74-7.78 (m, 1H), 6.24(s, 1H), 5.32-5.37 (m, 1H), 3.82 (s, 3H), 3.51-3.62 (m, 2H), 3.01-3.11 (m, 2H).

**Scheme 6.** Synthesis of compound 8

To a mixture of compound 7 (3.00 g, 8.10 mmol, 1.0 eq) in DCM (60 mL) was added BBr_3_ (10.2 g, 40.7 mmol, 5.0 eq) below 5 °C. The mixture stirred at room temperature for 3 h. The mixture was poured into water and then adjust pH to 8 with Na_2_CO_3_, and extracted with DCM (50 mL x 3). The combined organic phases were washed with brine, dried over Na_2_SO_4_ and concentrated in vacuum. The crude product was triturated with MTBE, filtered to give the compound 8 (2.00g, 69.4%) as yellow solid. (TLC: DCM/MEOH = 10/1, R_f_ = 0.5)

^1^H NMR (DMSO_d6, 400 MHz): δ 9.62 (brs, 1H), 9.16 (s, 1H), 8.57-8.77 (m, 2H), 8.69 (s, 1H), 8.53-8.55 (m, 1H), 8.40 (d, J = 8.0 Hz, 1H), 7.81 (d, J = 3.6 Hz, 2H), 7.52-7.56 (m, 1H), 7.06 (d, J = 8.0 Hz, 1H), 6.69 (s, 1H), 6.58-6.61 (m, 1H), 5.19-5.21 (m, 1H), 3.36-3.42 (m, 2H), 2.99-3.10 (m, 2H).

**Scheme 7.**  Synthesis of compound 9

To a mixture of compound 8 (2.00 g, 5.64 mmol, 1.0 eq) in MeCN (40 mL) was added compound 8A (from ATB-90, 330g, 6.71 mmol, 1.2 eq) and Cs_2_CO_3_ (3.70 g, 11.2 mmol, 2.0 eq). The mixture was heated to 80 °C and stirred overnight. The mixture was cooled to room temperature and poured into water and extracted with EA (50 mL x 3). The combined organic phases were washed with brine, dried over Na_2_SO_4_ and concentrated in vacuum. The residue was purified by silica gel, eluted with DCM/MeOH (1:0~100:1) to give the compound 9 (2.00 g, 52.6%) as yellow gel. (TLC: DCM/MeOH = 15/1, R_f_ = 0.4)

^1^H NMR (CDCl_3_, 400 MHz): δ 9.78 (s, 1H), 8.94 (s, 1H), 8.73 (s, 1H), 8.01 (s, 1H), 7.92 (s, 1H), 7.74-7.78 (m, 1H), 7.44-7.48 (m, 2H), 7.14-7.16 (d, J = 8.0 Hz, 1H), 6.76-6.80 (m, 2H), 5.31-5.37 (m, 1H), 5.14 (brs, 1H), 4.10-4.13 (m, 2H), 3.85-3.88 (s, 2H), 3.49-3.75 (m, 16H), 3.27-3.28 (m, 2H), 3.10-3.13 (m, 2H), 1.45 (s, 9H).

**Scheme 8.** Synthesis of compound 10

To a mixture of compound 9 (5.00 g, 7.42 mmol, 1.0 eq) in DCM (50 mL) was added dropwise TFA (15 mL) at 0 °C. The mixture was stirred for 3 h at room temperature. The mixture was poured into ice-water and then adjusted pH to 8 with NaHCO_3_, and extracted with DCM (100 mL x 3). The combined organic phases were dried over Na_2_SO_4_ and concentrated in vacuum to give the compound 10 (3.00 g, 70.5%) as yellow gel. (TLC: DCM/MEOH = 10/1, Rf = 0.4)

^1^H NMR (CDCl_3_, 400 MHz): δ 9.79 (s, 1H), 8.82-8.84 (m, 1H), 8.68-8.70 (m, 1H), 7.89-7.91 (m, 1H), 7.82-7.84 (m, 1H), 7.71-7.75 (m, 1H), 7.40-7.44 (m, 2H), 7.16 (d, J = 8.4 Hz, 1H), 6.86 (s, 1H), 6.77-6.79 (m, 1H), 6.40 (d, J = 6.4 Hz, 1H), 5.30-5.32 (m, 1H), 4.11-4.14 (m, 2H), 3.84-3.87 (m, 2H), 3.48-3.77 (m, 16H), 3.00-3.32 (m, 5H), 2.85-2.87 (m, 2H).

**Scheme 9.** Synthesis of ATB-152

A mixture of compound 10 (3.00 g, 5.23 mmol, 1.0 eq) and compound 10A (from ATB-134, 2.50 g, 6.27 mmol, 1.2 eq) in MeCN (30 mL) was heated to 80 °C and stirred overnight. The mixture was concentrated. The residue was purified by silica gel, eluted with DCM/MeOH (1:0 to 40:1) to give ATB-152 (1.40 g, 27.6%) as yellow solid. (TLC: DCM/MEOH = 10/1, Rf = 0.4)

^1^H NMR (DMSO_d6, 400 MHz): δ 9.63 (s, 1H), 8.75 (dd, J = 1.6, 8.0 Hz, 1H), 8.67 (dd, J = 1.6, 4.8 Hz, 1H), 8.49 (d, J = 6.0 Hz, 1H), 8.39 (d, J = 8.0 Hz, 1H), 7.77-7.82 (m, 2H), 7.41-7.53 (m, 6H), 7.15-7.23 (m, 5H), 6.88-6.92 (m, 2H), 6.76 (dd, J = 2.4, 8.0 Hz, 1H), 6.67 (d, J = 2.8 Hz, 1H), 3.77-3.86 (m, 3H), 3.71-3.74 (m, 2H), 3.37-3.60 (m, 18H), 3.02-3.15 (m, 2H), 2.66-2.72 (m, 3H), 2.58-2.63 (m, 1H).

**Scheme 10.** Synthesis of ATB2057 & ATB2058

ATB-152 (1.4 g) was purified by prep-chiral HPLC (Gilson, Column: Chiralpak IB 250mm * 20mm 5 μm, Mobile Phase: MeOH: EtOH: DEA=50: 50: 0.2, Flow Rate: 15 mL/min, Detector Wavelength: 254 nm, P1 ~ 12.2 min, P2 ~ 16.8 min) to give ATB2057 (560 mg) as yellow solid and ATB2058 (580 mg) as yellow solid.

**ATB2057:**

^1^H-NMR (DMSO_d6, 400 MHz): δ 9.63 (s, 1H), 8.75 (dt, J = 1.6, 8.0 Hz, 1H), 8.68(dd, J = 1.6, 4.8 Hz, 1H), 8.39 (d, J = 8.0 Hz, 1H), 7.79-7.81 (m, 2H), 7.41-7.53 (m, 6H), 7.15-7.23 (m, 5H), 6.88-6.92 (m, 2H), 6.76 (dd, J = 2.4, 8.0 Hz, 1H), 6.67 (d, J = 2.8 Hz, 1H), 6.42 (dd, J = 2.8, 8.8 Hz, 1H), 5.18-5.21 (m, 1H), 5.08 (s, 2H), 4.98 (m, 2H), 4.05-4.07 (m, 2H), 3.79-3.86 (m, 3H), 3.71-3.74 (m, 2H), 3.35-3.60 (m, 18H), 3.04-3.15 (m, 2H), 2.66-2.72 (m, 3H), 2.58-2.63 (m,1H).

LC-MS [mobile phase: from 90% water (0.1% TFA) and 10% CH_3_CN to 5 % water (0.1% TFA) and 95% CH_3_CN in 6.0 min, finally under these conditions for 0.5 min.] purity is >98%, Rt = 2.874 min; Mass Calcd.:971; MS Found: 972[MS+1], 486[MS/2+1].

**ATB2058:**

^1^H-NMR (DMSO_d6, 400 MHz): δ 9.63 (s, 1H), 8.75 (dt, J = 2.0, 8.0 Hz, 1H), 8.67(dd, J = 1.6, 4.8 Hz, 1H), 8.49 (d, J = 6.8 Hz, 1H), 8.39 (d, J= 8.4 Hz, 1H), 7.79-7.81 (m, 2H), 7.41-7.53 (m, 6H), 7.15-7.23 (m, 5H), 6.88-6.92 (m, 2H), 6.76 (dd, J = 2.4, 8.0 Hz, 1H), 6.67 (d, J = 2.8 Hz, 1H), 6.42 (dd, J = 2.8, 8.8 Hz, 1H), 5.16-5.24 (m, 1H), 5.08 (s, 2H), 4.98 (s, 2H), 4.05-4.07 (m, 2H), 3.77-3.85 (m, 3H), 3.71-3.74 (m, 2H), 3.37-3.60 (m, 18H), 3.02-3.15 (m, 2H), 2.65-2.74 (m, 3H), 2.56-2.62 (m,1H).

LC-MS [mobile phase: from 90% water (0.1% TFA) and 10% CH_3_CN to 5 % water (0.1% TFA) and 95% CH_3_CN in 6.0 min, finally under these conditions for 0.5 min.] purity is >98%, Rt = 2.875 min; Mass Calcd.:971; MS Found: 972[MS+1], 486[MS/2+1].

**Supplemental Figures**


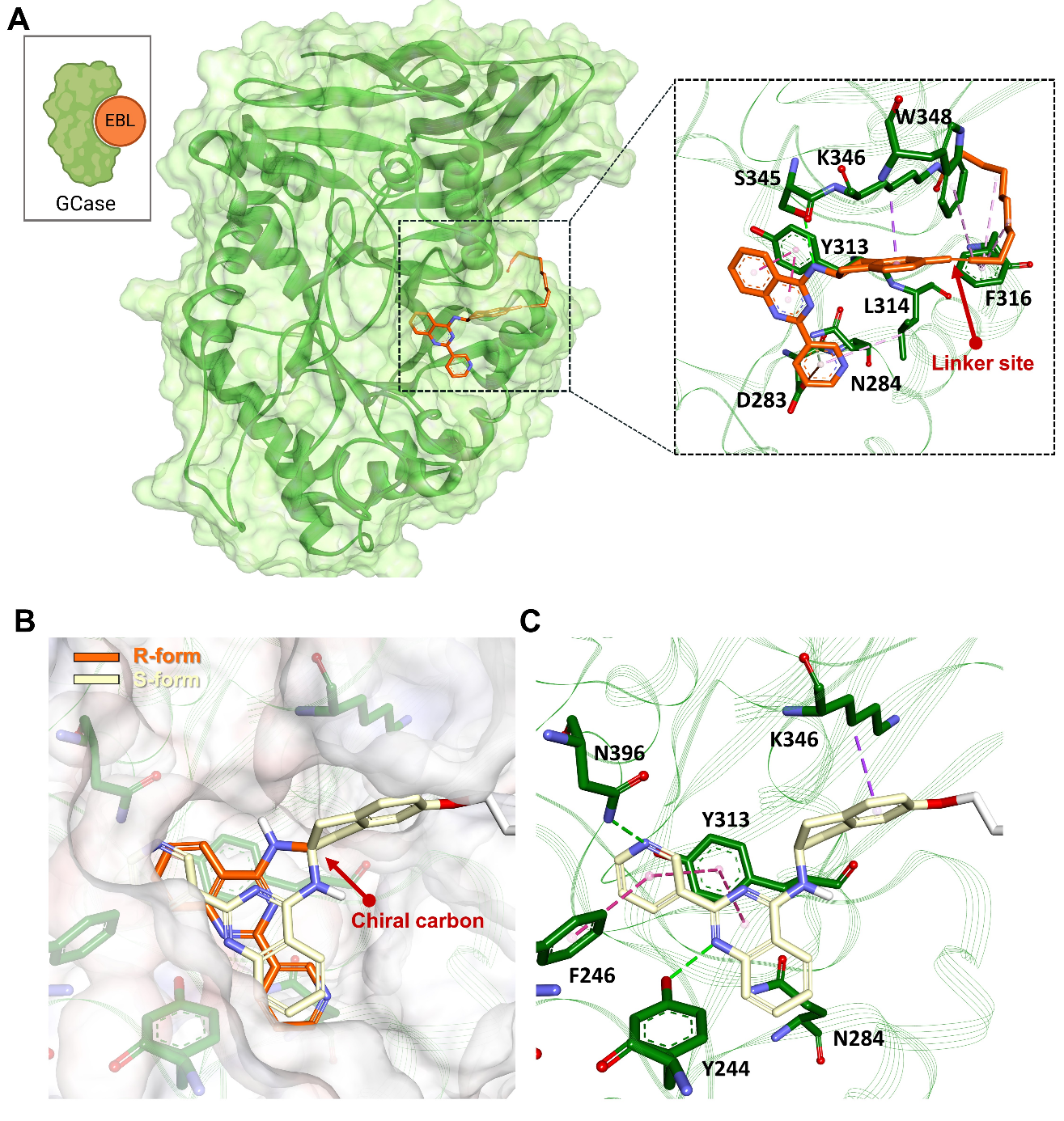


**Fig. S1.** **Structural comparison of EBL R- and S-isomers in the GCase active site. (A)** Crystal structure of GCase in complex with JZ-4109 (PDB ID: 5LVX). GCase is represented in green (cartoon and surface), and JZ-4109 is shown as an orange stick model. The linker attachment site is indicated by a red arrow. The cartoon in the panel with a solid border was created in BioRender. Park, J. (2026) https://BioRender.com/xjve2jf. **(B)** Superimposed conformations of EBL R-form and S-form, shown as orange and pale-yellow sticks, respectively, within the GCase binding pocket surface. The chiral carbon is indicated by a red arrow. **(C)** Binding interactions of the EBL S-form with key residues in the GCase active site are depicted by dashed lines, with green indicating hydrogen bonds, pink indicating π-π interactions, and purple indicating π-alkyl interactions.

**
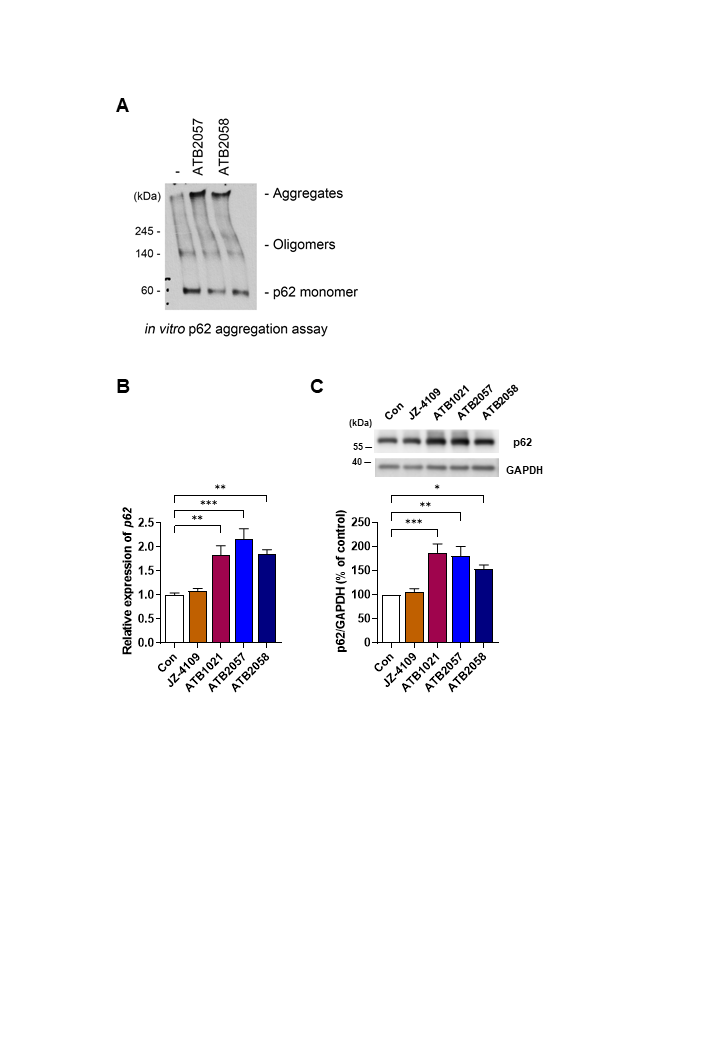
**

**Fig. S2.** **LYSOTAC increases p62 expression. (A)** *In vitro* p62 aggregation assay. **(B)** Relative *p62* mRNA levels and **(C)** p62 protein levels in HeLa cells treated with the indicated compounds (2 μM, 1 day) (*n* = 6). Differences between groups were evaluated by one-way ANOVA. The data are presented as the mean ± SEM. **p*<0.05, ***p*<0.01, ****p*<0.001.

**
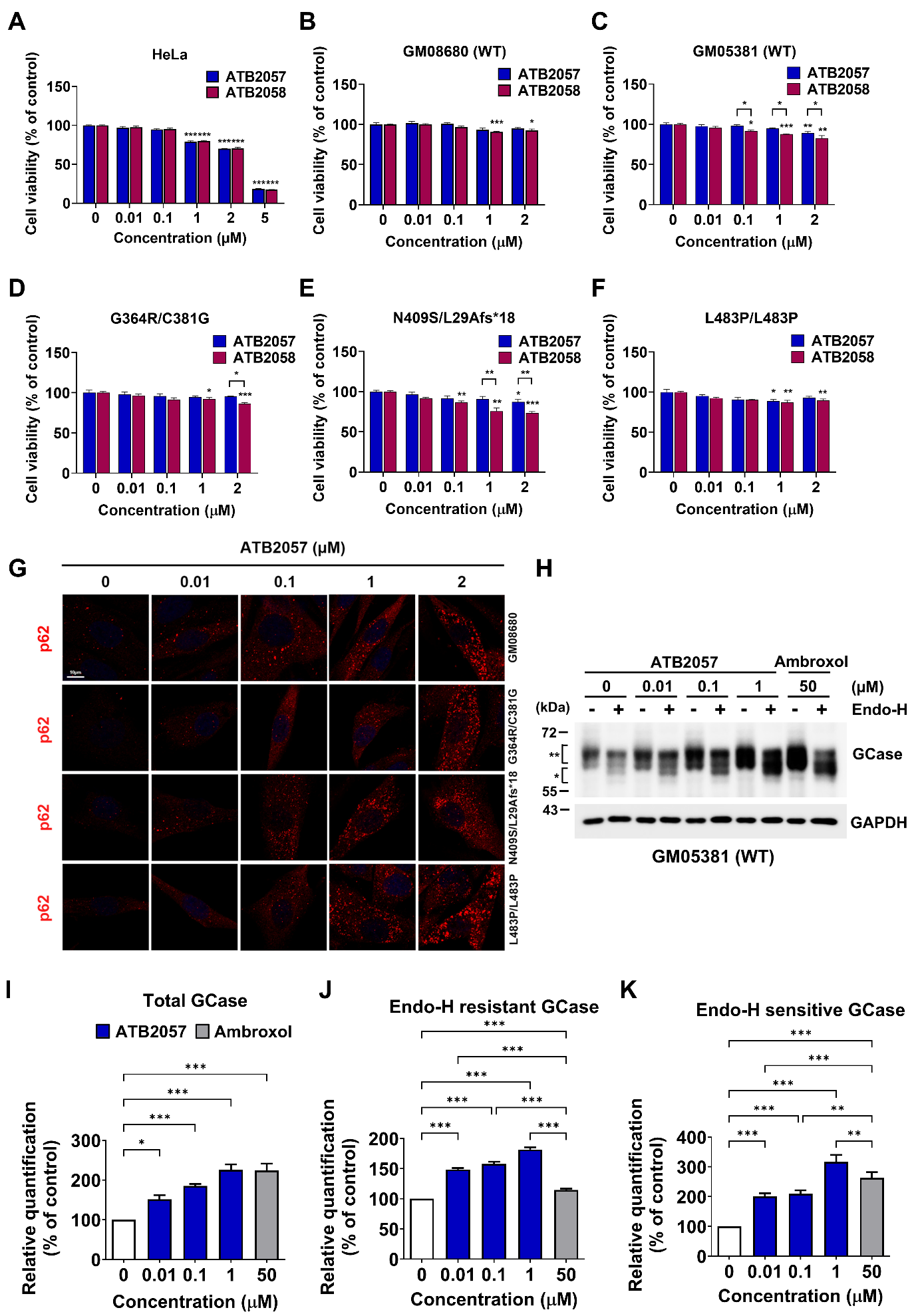
**

**Fig. S3. Cell viability and p62 oligomerization are influenced by LYSOTAC concentration.** Cell viability of **(A)** HeLa cells, WT fibroblasts (**(B)** GM08680, **(C)** GM05381), and Gaucher disease (GD) fibroblasts (**(D)** GM02627 [G364R/C381G], **(E)** GM00372 [N409S/L29Afs*18], and **(F)** GM07968 [L483P/L483P]) treated with ATB2057 or ATB2058 for 24 h were measured using MTT assay (n = 5). **(G)** Immunocytochemistry (ICC) of p62 puncta in fibroblasts treated with ATB2057 for 24 h. **(H)** Lysates were subjected to Endo-H digestion, and the Endo-H resistant and sensitive fractions are marked by ** and *, respectively. Western blotting (WB) of normal human fibroblasts (GM05381) treated with ATB2057 or ambroxol (50 μM) for 10 days. Quantification of **(I)** total GCase, **(J)** Endo-H resistant GCase, and **(K)** Endo-H sensitive GCase of (H) (*n* = 5). Differences between groups were evaluated by two-way ANOVA. The data are presented as the mean ± SEM.**p*<0.05, ***p*<0.01, ****p*<0.001.

**
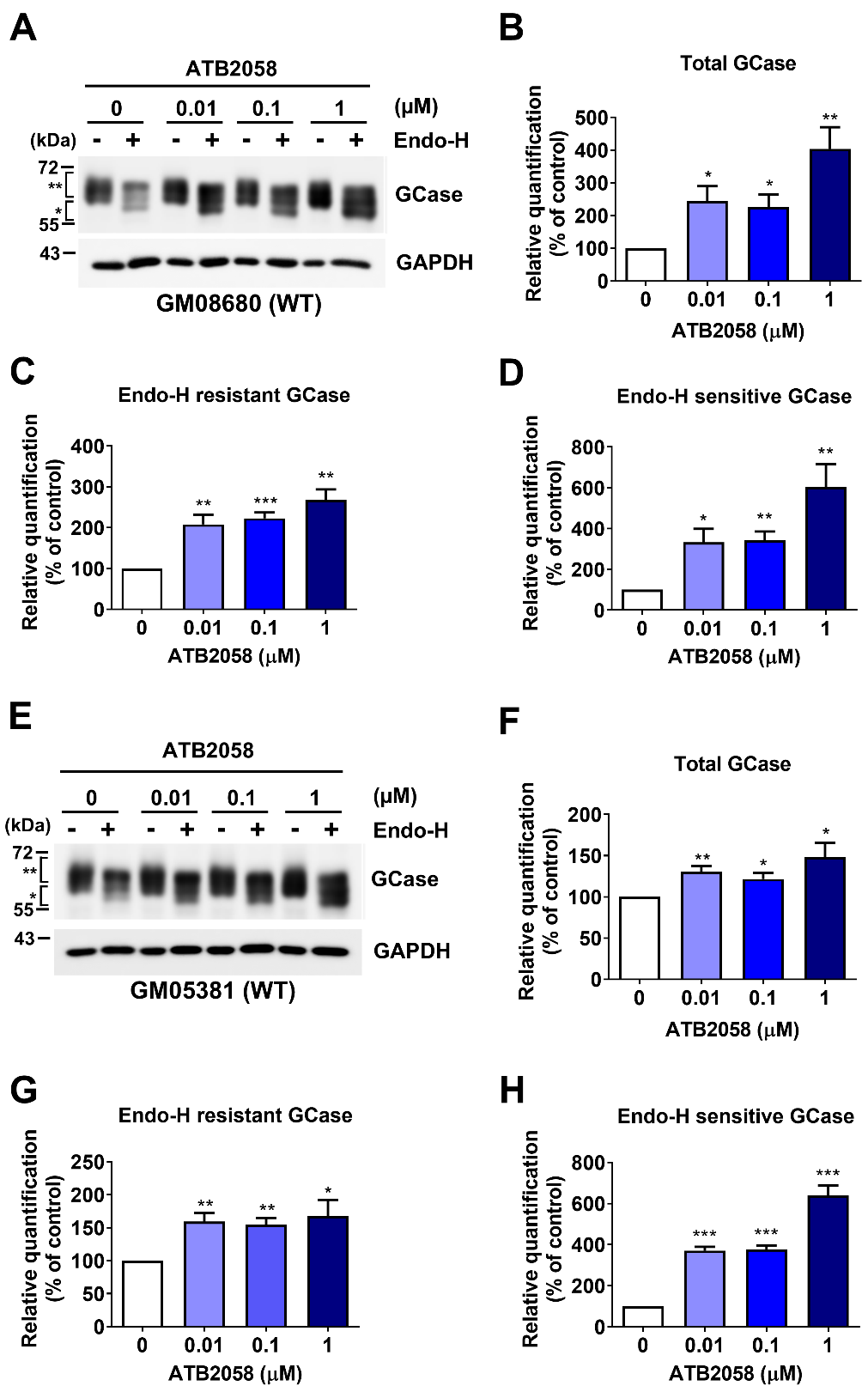
**

**Fig. S4. ATB2058 increases GCase protein levels in WT human fibroblasts.** Lysates were subjected to Endo-H digestion and the Endo-H resistant and sensitive fractions are marked by ** and *, respectively. **(A)** WB of normal human fibroblasts (GM08680) treated with ATB2058 for 10 days. Quantification of **(B)** total GCase, **(C)** Endo-H resistant GCase, and **(D)** Endo-H sensitive GCase of (A) (*n* = 5). **(E)** WB of normal human fibroblasts (GM05381) treated with ATB2058 for 10 days. Quantification of **(F)** total GCase, **(G)** Endo-H resistant GCase, and **(H)** Endo-H sensitive GCase of (E) (*n* = 5). Differences between groups were evaluated by one-way ANOVA. The data are presented as the mean ± SEM. **p*<0.05, ***p*<0.01, ****p*<0.001.


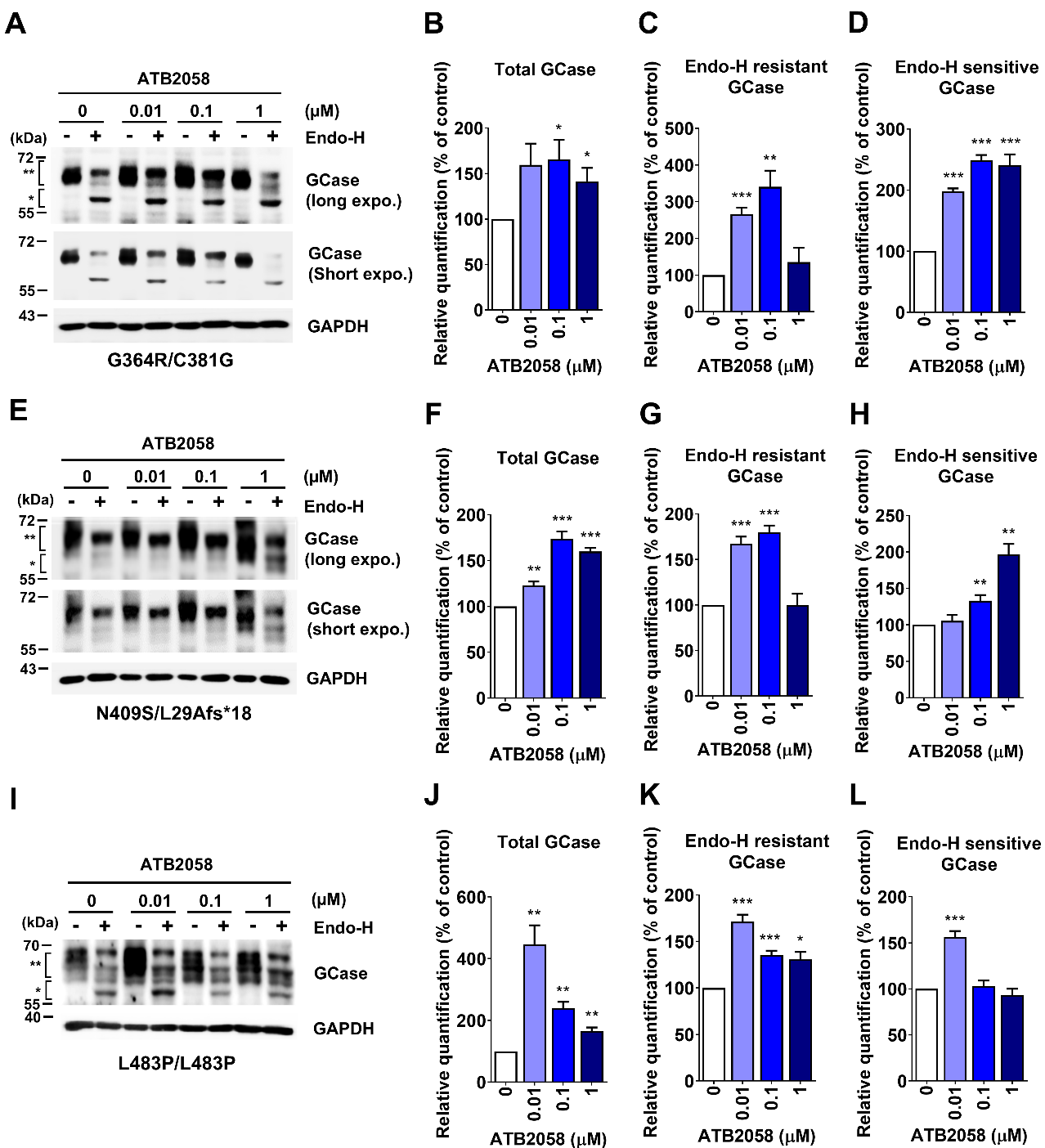


**Fig. S5. ATB2058 restores GCase protein levels in GD fibroblasts.** Lysates were subjected to Endo-H digestion and the Endo-H resistant and sensitive fractions are marked by ** and *, respectively. **(A)** WB of human GD fibroblasts (GM02627) treated with ATB2058 for 10 days. Quantification of **(B)** total GCase, **(C)** Endo-H resistant GCase, and **(D)** Endo-H sensitive GCase of (A) (*n* = 5). **(E)** WB of human GD fibroblasts (GM00372) treated with ATB2058 for 10 days. Quantification of **(F)** total GCase, **(G)** Endo-H resistant GCase, and **(H)** Endo-H sensitive GCase of (E) (*n* = 5). **(I)** WB of human GD fibroblasts (GM07968) treated with ATB2058 for 10 days. Quantification of **(J)** total GCase, **(K)** Endo-H resistant GCase, and **(L)** Endo-H sensitive GCase of (I) (*n* = 5). Differences between groups were evaluated by one-way ANOVA. The data are presented as the mean ± SEM. **p*<0.05, ***p*<0.01, ****p*<0.001.

**
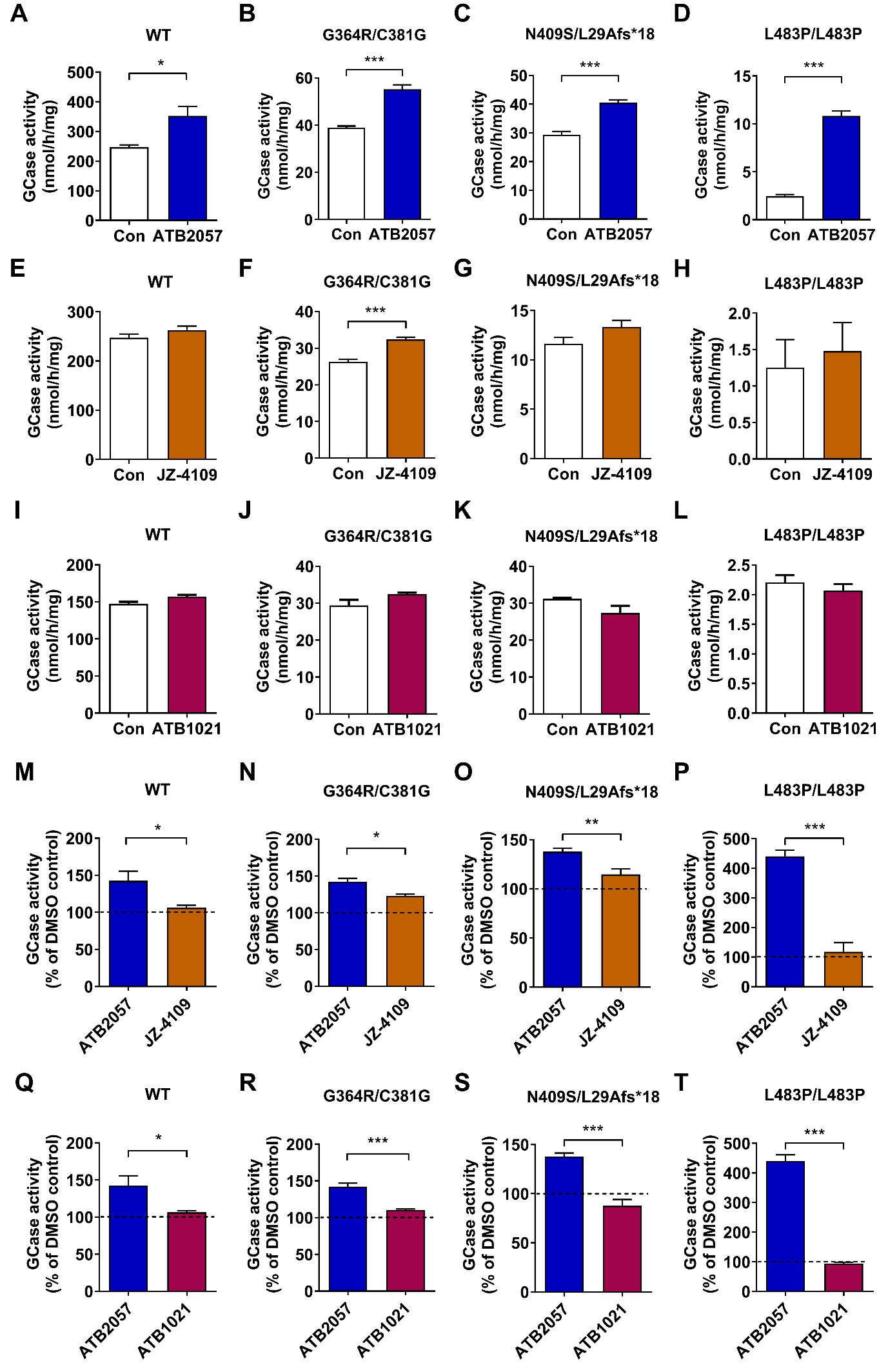
**

**Fig. S6. GCase activity is increased by ATB2057 in both WT and GD fibroblasts.** GCase activity in **(A)** WT (GM08680) and GD fibroblasts (**(B)** GM02627, **(C)** GM00372, and **(D)** GM07968) treated with ATB2057 (0.1 μM) for 10 days. GCase activity in **(E)** WT (GM08680) and GD fibroblasts (**(F)** GM02627, **(G)** GM00372, and **(H)** GM07968) treated with JZ-4109 (0.1 μM) for 10 days. GCase activity in **(I)** WT (GM08680) and GD fibroblasts (**(J)** GM02627, **(K)** GM00372, and **(L)** GM07968) treated with ATB1021 (0.1 μM) for 10 days. Data are displayed as raw data (*n* = 5). GCase activity in **(M)** WT (GM08680) and GD fibroblasts (**(N)** GM02627, **(O)** GM00372, and **(P)** GM07968) treated with ATB2057 (0.1 μM) or JZ-4109 (0.1 μM) is represented as the percentage of DMSO-treated control (*n* = 5). GCase activity in **(M)** WT (GM08680) and GD fibroblasts (**(N)** GM02627, **(O)** GM00372, and **(P)** GM07968) treated with ATB2057 (0.1 μM) or ATB1021 (0.1 μM) is represented as the percentage of DMSO-treated control (*n* = 5). Differences between groups were evaluated by unpaired Student’s *t*-test. The data are presented as the mean ± SEM. **p*<0.05, ***p*<0.01, ****p*<0.001.


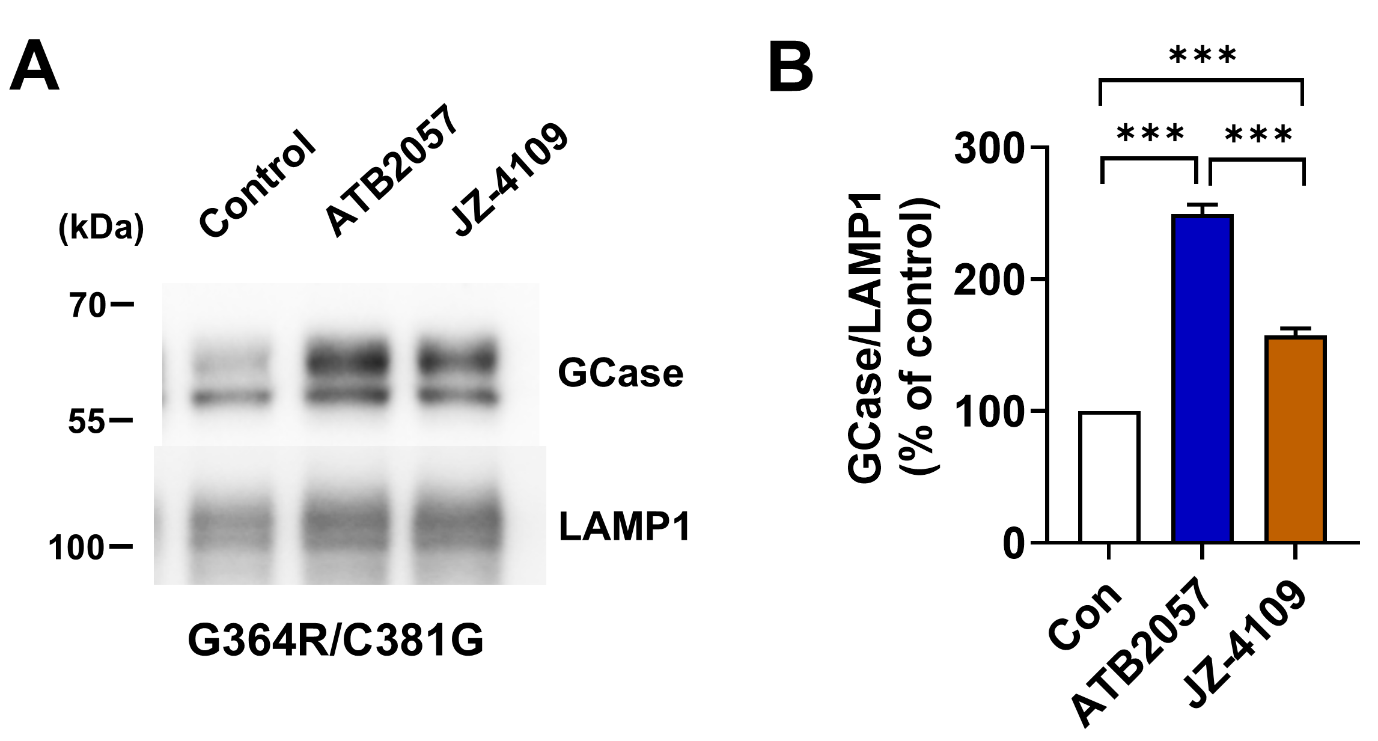


**Fig. S7. ATB2057 enhances lysosomal delivery of GCase compared with JZ-4109.** Lysosomal fractions were isolated from GD fibroblasts (GM02627) following treatment with ATB2057 or JZ-4109 (0.1 μM, 2 days). **(A)** Representative WB and **(B)** quantification of GCase levels normalized to LAMP1 are displayed (n = 5). Statistical significance was assessed by one-way ANOVA. The data are presented as the mean ± SEM. ***p<0.001.


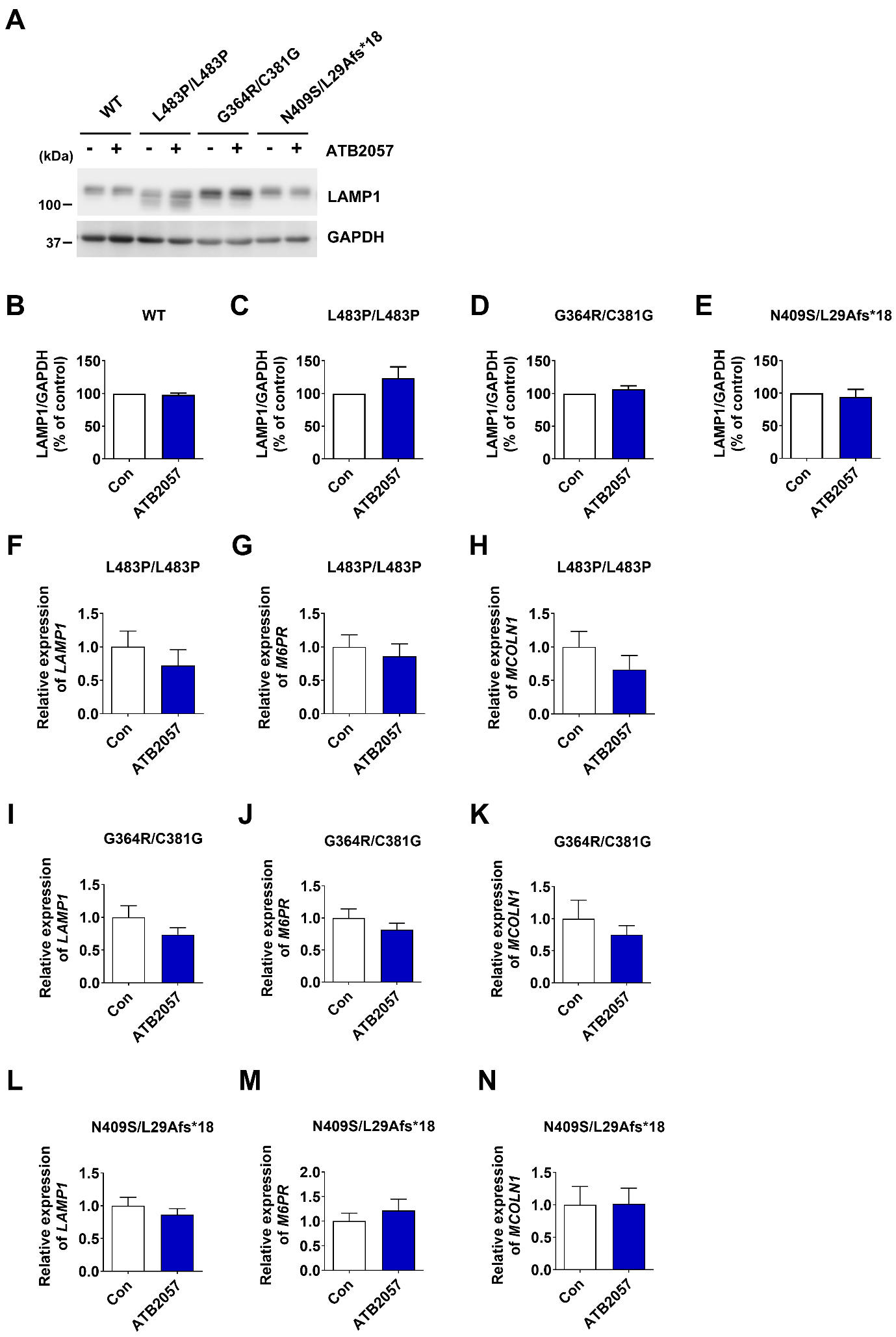


**Fig. S8. ATB2057 does not induce lysosomal biogenesis in WT and GD fibroblasts. (A)** WB of LAMP1 in human WT (GM08680) and GD fibroblasts (GM07968, GM02627, GM00372) treated with ATB2057 (0.1 μM, 2 days). Quantification of LAMP1 for the corresponding cell lines (*n* = 6): **(B)** WT, **(C)** GM07968, **(D)** GM02627, and **(E)** GM00372. **(F-N)** Relative mRNA levels of lysosomal biogenesis–related genes (*LAMP1*, *M6PR*, and *MCOLN1*) analyzed in GD fibroblasts treated with ATB2057 (0.1 μM, 2 days) (n = 6). Data are mean ± SEM. Differences were not statistically significant.


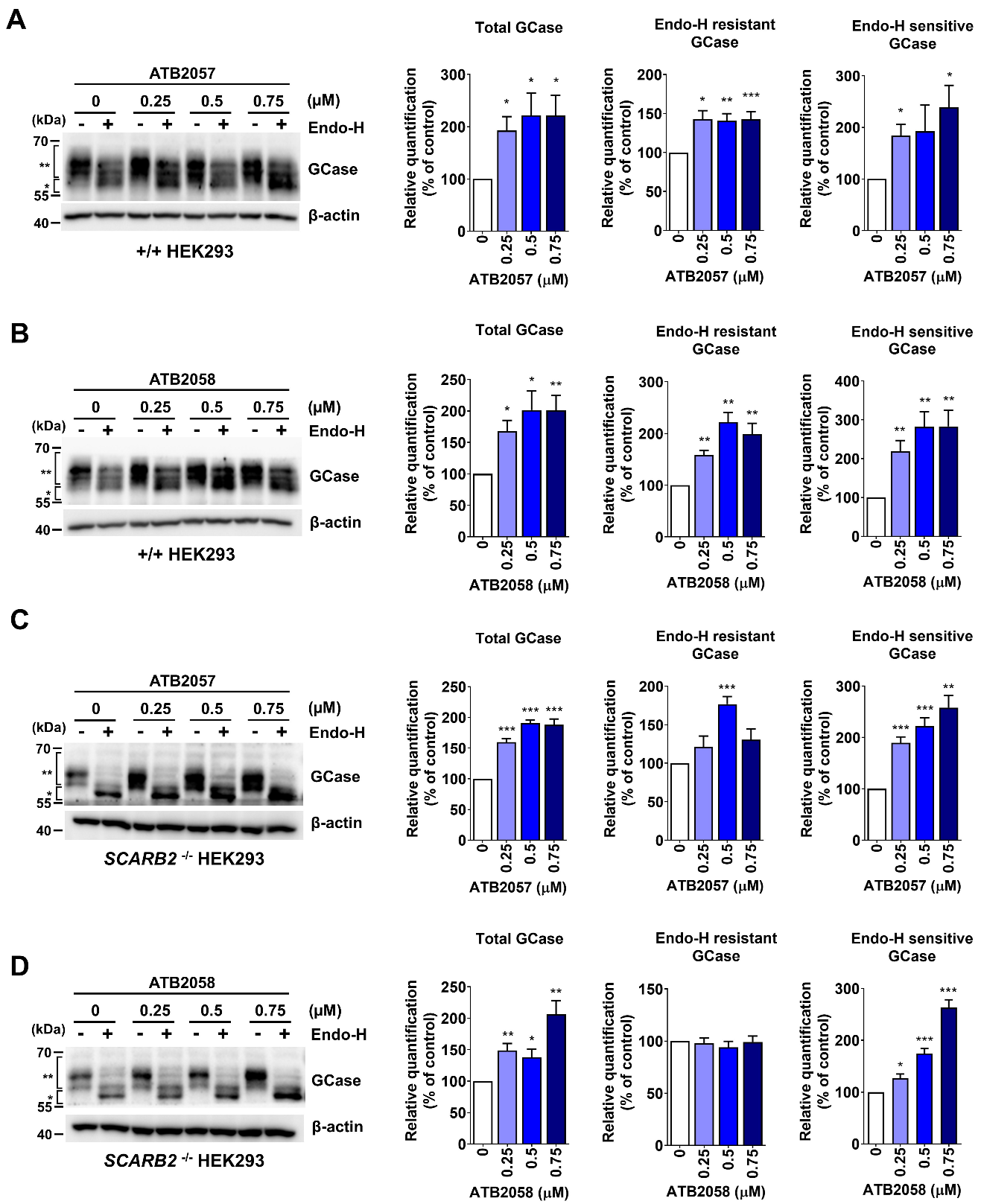


**Fig. S9. GCase protein levels are restored in LIMP-2 deficient cells mainly composed of Endo-H sensitive form.** Lysates were subjected to Endo-H digestion and the Endo-H resistant and sensitive fractions are marked by ** and *, respectively. WB of control HEK293 cells incubated with **(A)** ATB2057 or **(B)** ATB2058 for 7 days. WB of LIMP-2 deficient HEK293 cells incubated with **(C)** ATB2057 or **(D)** ATB2058 for 7 days. Quantification of total, Endo-H resistant, and Endo-H sensitive GCase are presented (*n* = 5). Differences between groups were evaluated by one-way ANOVA. The data are presented as the mean ± SEM. **p*<0.05, ***p*<0.01, ****p*<0.001.

**
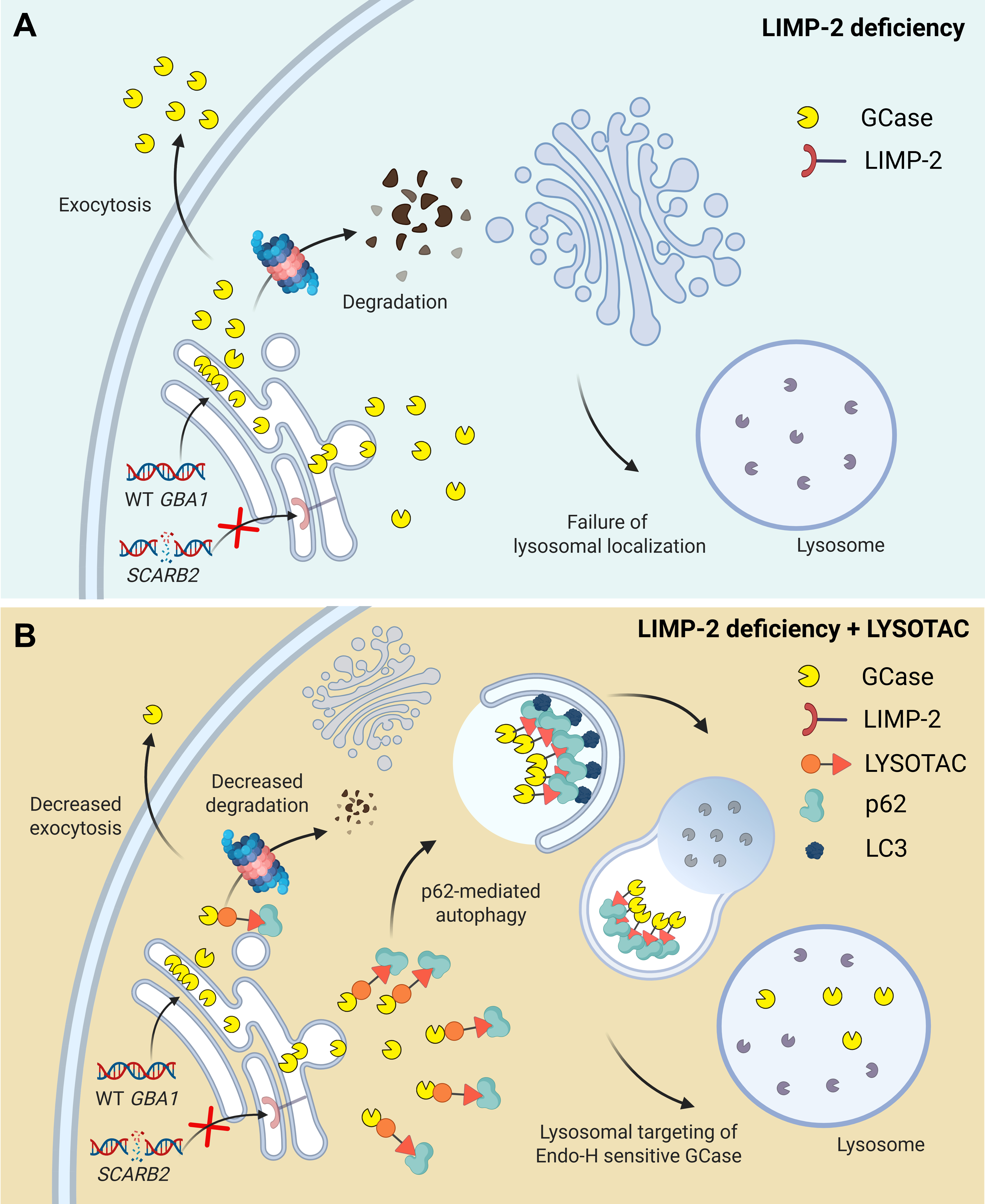
**

**Fig. S10. LYSOTAC improves lysosomal delivery of GCase, mostly Endo-H sensitive form, under LIMP-2 deficiency. (A)** Schematic of intracellular GCase processing under LIMP-2 deficiency. **(B)** Schematic of GCase processing applied with LYSOTAC in LIMP-2 deficient cells. Created in BioRender. Park, J. (2026) https://BioRender.com/wduhe6c.

**
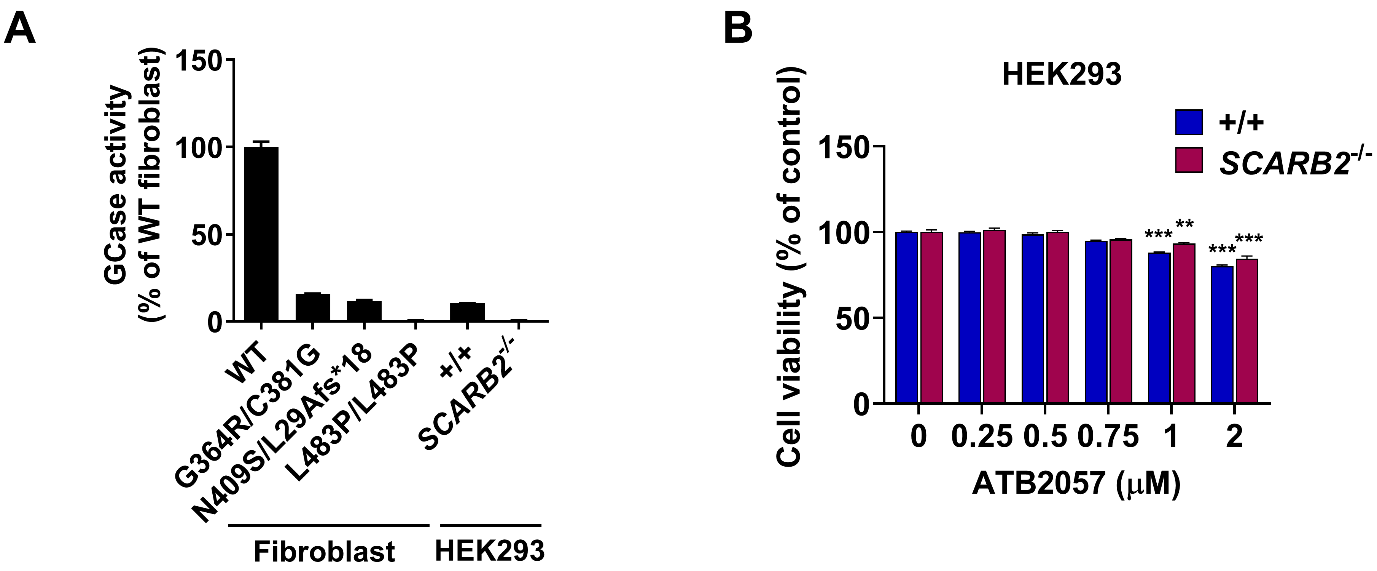
**

**Fig. S11. Cell type–dependent variation in GCase activity. (A)** Basal GCase activity in the indicated cells (n = 6). **(B)** Viability of HEK293 cells following 24 h treatment with ATB2057, measured by MTT assay (n = 6). Differences between groups were analyzed by one-way ANOVA. The data are presented as the mean ± SEM. ***p*<0.01, ****p*<0.001.

**
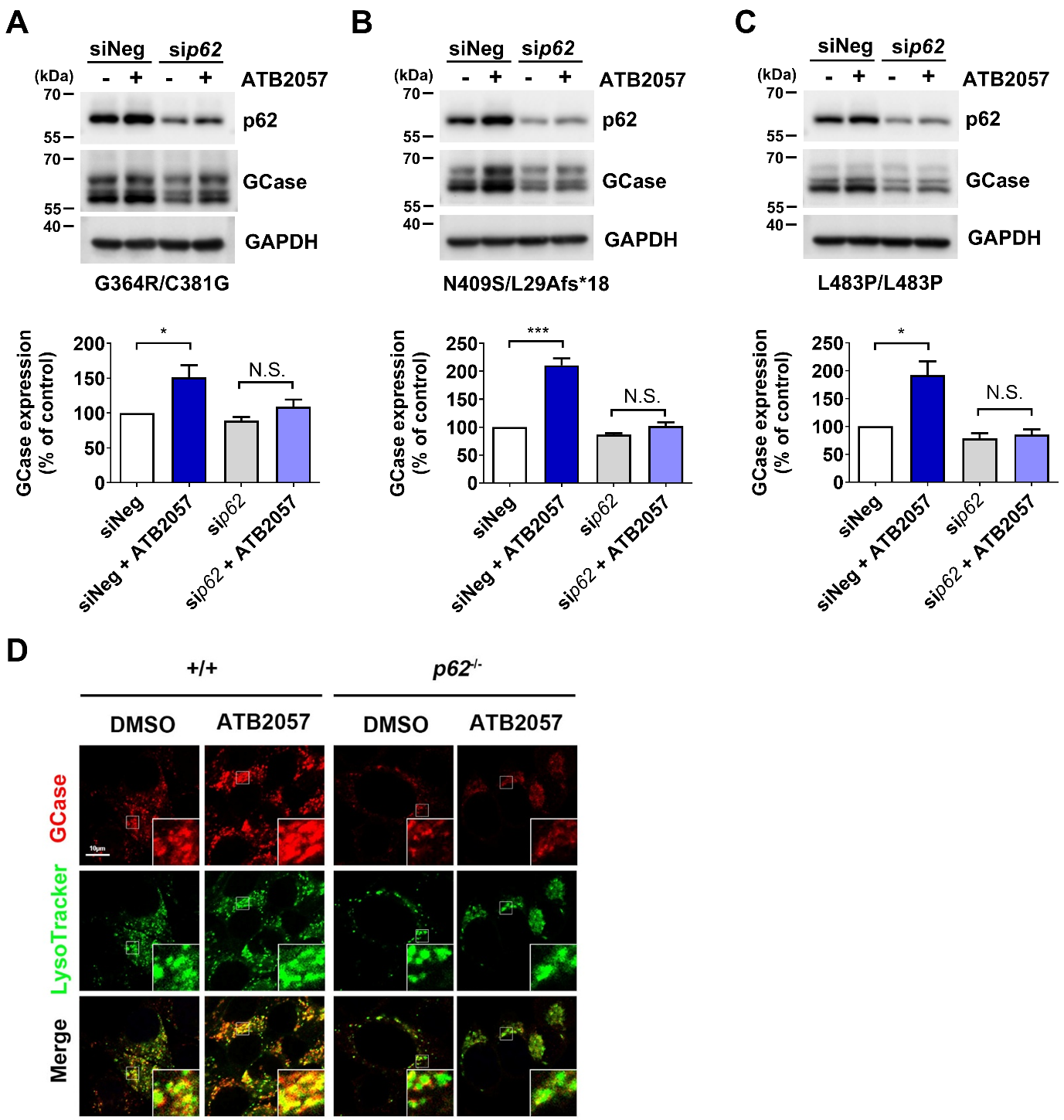
**

**Fig. S12. ATB2057 improves lysosomal GCase delivery via p62.** WB of human GD fibroblasts carrying *GBA1* **(A)** G364R/C381G, **(B)** N409S/ L29Afs*18, or (**C)** L483P/L483P mutations treated with combination of ATB2057 (0.1 μM) and siRNA for p62 (si*p62*) (*n* = 6). **(D)** ICC of GCase (Alexa Fluor 647) with LysoTracker staining (DND-99, pseudo-colored green) in control and *p62*^-/-^ HeLa cells treated with ATB2057 (1 μM) or its vehicle, DMSO for 24 h. Differences between groups were evaluated by two-way ANOVA. The data are presented as the mean ± SEM. **p*<0.05, ****p*<0.001, N.S. not significant.

**Table of Contents**

- Appendix Figure S1. Chemical synthesis scheme of LYSOTACs
- Appendix Figure S2. ^1^H-NMR spectrum of LYSOTACs
- Appendix Figure S3. LC-MS data of LYSOTACs

**Appendix Figure S1. Chemical synthesis scheme of LYSOTACs**

**
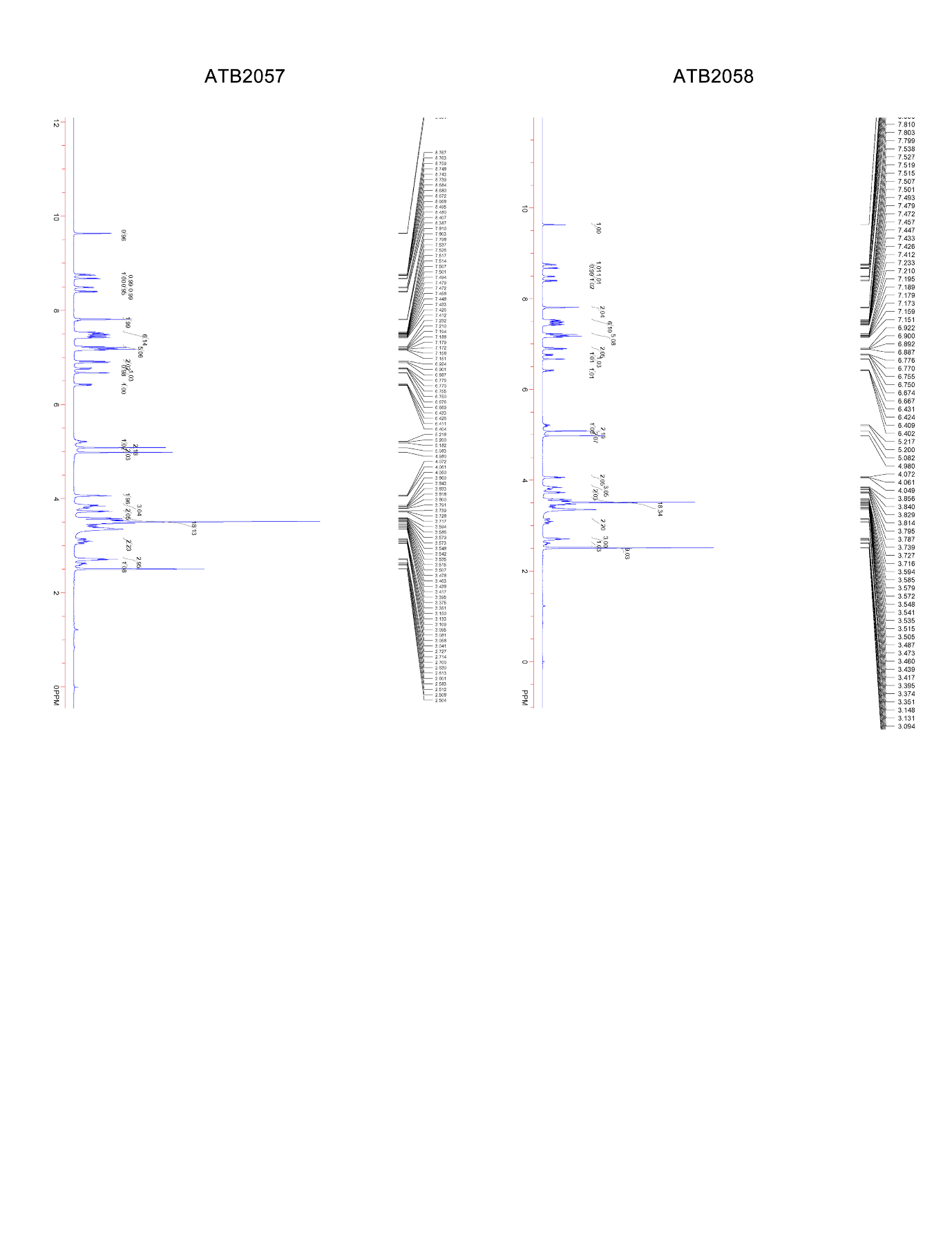
**

**Appendix Figure S2. ^1^H-NMR spectrum of LYSOTACs**

**
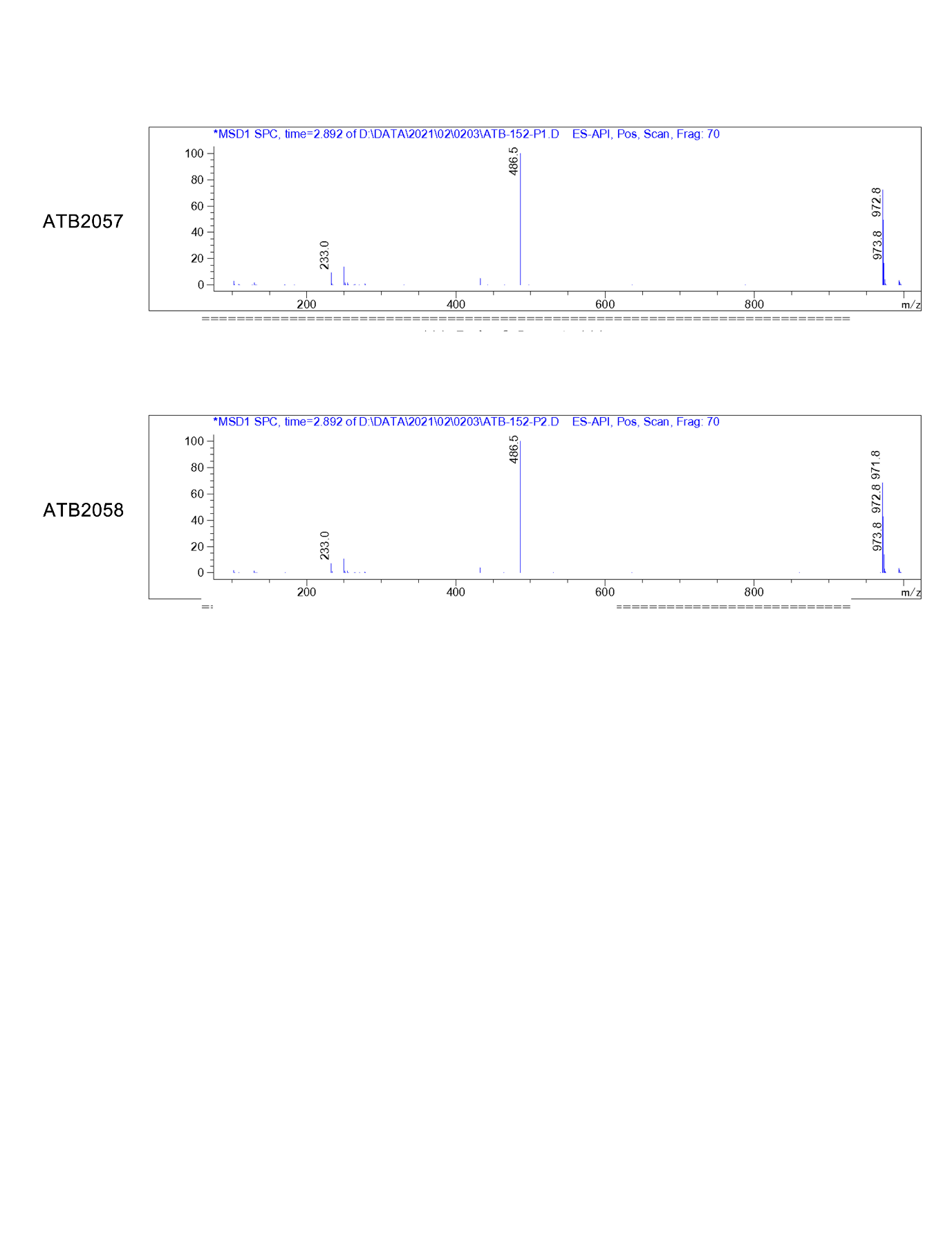
**

**Appendix Figure S3. LC-MS data of LYSOTACs**
